# Prognostic implications of troponin T variations in inherited cardiomyopathies using systems biology

**DOI:** 10.1038/s41525-021-00204-w

**Published:** 2021-06-14

**Authors:** Rameen Shakur, Juan Pablo Ochoa, Alan J. Robinson, Abhishek Niroula, Aneesh Chandran, Taufiq Rahman, Mauno Vihinen, Lorenzo Monserrat

**Affiliations:** 1 The Koch Institute for Integrative Cancer Research, Massachusetts Institute of Technology, 500 Main Street, Boston, Massachusetts 02459 United States; 2 Wellcome Trust Sanger Institute, Wellcome Trust Genome Campus, Hinxton, CB10 1RQ UK; 3 Institute of Biomedical Investigation of A Coruña (INIBIC), University of A Coruña, Hospital Marítimo de Oza (15006), A Coruña, Spain; 4 Cardiology department, Health In Code. As Xubias s/n, Edificio El Fortín, 15006 A Coruña, Spain; 5 Medical Research Council Mitochondrial Biology Unit, The Keith Peters Building, Cambridge Biomedical Campus, Hills Road, Cambridge, CB2 0XY UK; 6 Protein Structure and Bioinformatics, Department of Experimental Medical Science, Lund University, SE-22 184 Lund, Sweden; 7 Department of Biotechnology & Microbiology, Kannur University, Kannur, 670 661 Kerala India; 8 Department of Pharmacology, University of Cambridge, Cambridge, CB2 1PD UK

**Keywords:** Genetics research, Systems biology

## Abstract

The cardiac troponin T variations have often been used as an example of the application of clinical genotyping for prognostication and risk stratification measures for the management of patients with a family history of sudden cardiac death or familial cardiomyopathy. Given the disparity in patient outcomes and therapy options, we investigated the impact of variations on the intermolecular interactions across the thin filament complex as an example of an unbiased systems biology method to better define clinical prognosis to aid future management options. We present a novel unbiased dynamic model to define and analyse the functional, structural and physico-chemical consequences of genetic variations among the troponins. This was subsequently integrated with clinical data from accessible global multi-centre systematic reviews of familial cardiomyopathy cases from 106 articles of the literature: 136 disease-causing variations pertaining to 981 global clinical cases. Troponin T variations showed distinct pathogenic hotspots for dilated and hypertrophic cardiomyopathies; considering the causes of cardiovascular death separately, there was a worse survival in terms of sudden cardiac death for patients with a variation at regions 90–129 and 130–179 when compared to amino acids 1–89 and 200–288. Our data support variations among 90–130 as being a hotspot for sudden cardiac death and the region 131–179 for heart failure death/transplantation outcomes wherein the most common phenotype was dilated cardiomyopathy. Survival analysis into regions of high risk (regions 90–129 and 130–180) and low risk (regions 1–89 and 200–288) was significant for sudden cardiac death (*p* = 0.011) and for heart failure death/transplant (*p* = 0.028). Our integrative genomic, structural, model from genotype to clinical data integration has implications for enhancing clinical genomics methodologies to improve risk stratification.

## Introduction

The most common forms of genetic heart disease are the inherited cardiomyopathies, which affect ~0.2% of the global population^1,2^. The cardiomyopathies are a group of rare heart muscle disorders that afflict the structure and physiological function of the myocardium. The two most common traditional pathological forms are dilated and hypertrophic cardiomyopathies, each with characteristic clinical phenotypes and often showing an autosomal dominant inheritance. The most common genetic variations appear in sarcomeric proteins; of which the troponin (Tn) proteins are part of the larger thin filament complex within the regulatory unit of the sarcomere. The Tn variations are thought to contribute approximately to 8–10% of all the known sarcomeric protein cardiomyopathies^3^. However, given the lack of a complete Tn complex, analysis has often been limited to sub-complexes. Although there are many Electron Microscopy and Nuclear magnetic resonance interaction data, the resolution is too limited to provide a protein–protein interaction map.

The Tns are a complex of three subunits: Tn I (TnI) inhibits actomyosin ATPase; Tn C (TnC) binds calcium; and Tn T (TnT) links the complex to tropomyosin (Tm) and is believed to be responsible for the movement of Tm on the thin filament, modulating binding of the myosin head to actin. The subunits are arranged in a 1:1:1 stoichiometric ratio along the thin filament with one Tn: Tm complex bound to every seven actin monomers^4^.

Since the first observations associating genetic variations in the Tn complex to morphological classifications—such as hypertrophic cardiomyopathy (HCM) and an increased risk of sudden cardiac death (SCD)—there have been many studies attempting to define the clinical prognostic and management implications of genotype-phenotype conundrums^5–7^. Most variations are related to HCM, although others may cause dilated cardiomyopathy (DCM) and less frequently restrictive cardiomyopathies (RCM) with differing clinical outcomes^8^. For example, variations in the cardiac TnT gene (*TNNT2*) cause HCM with variable clinical phenotypes^7,9^. Some patients with these variations have a high risk of ventricular arrhythmias and SCD, even with little or no left ventricular hypertrophy to surmise prognosis^7^. This poses difficulty on the optimum timing for the application of device therapy (such as implantable cardiac defibrillators) for patients, and so many have tried to determine if genetic prognostication can help^1^. Early comparisons of two TnT mutants-one associated with HCM, and the other with DCM-noted qualitatively different functional consequences of the TnT variations on calcium sensitivity, ATPase activity and sliding speed, and concluded this led to divergent phenotypes of HCM and DCM^10^.

Therefore, the application of clinical genomics has often been hampered due to the lack of integrated and unbiased representation of the genomic, structural and clinical phenotypic interplay, whilst trying to grapple with ascertainment bias^1^. However, the ideal for a structural analysis would be a fully co-crystallised high-resolution structure of full-length F-actin-Tm-Tn complex. However, such a structure is currently unavailable. These observations underlie the longstanding complexity between genotype-phenotype correlations in real-world clinical practice. Hence, a prognostic model must account for the dynamic nature of cardiac contraction in the genomic landscape in regards to possible phenotype-specific hotspots and provide insights on thin filament variations and cardiomyopathies and their potential clinical sequelae.

Given the disparity in patient outcomes and therapy options, and the potential for exploiting genotype-phenotype implications to improve patient care, we investigated the impact of variations on the intermolecular interactions across the thin filament complex. Our findings are pertinent to better define and instigate the application of clinical genomic data for inherited diseases, such as the cardiac TnT variations, and how in high-risk categories we may best stratify risk and define potential invasive or non-invasive therapy.

## Results

### Intermolecular interactions across the troponins are dynamic and dependent on the calcium state

**Fig. 1:**
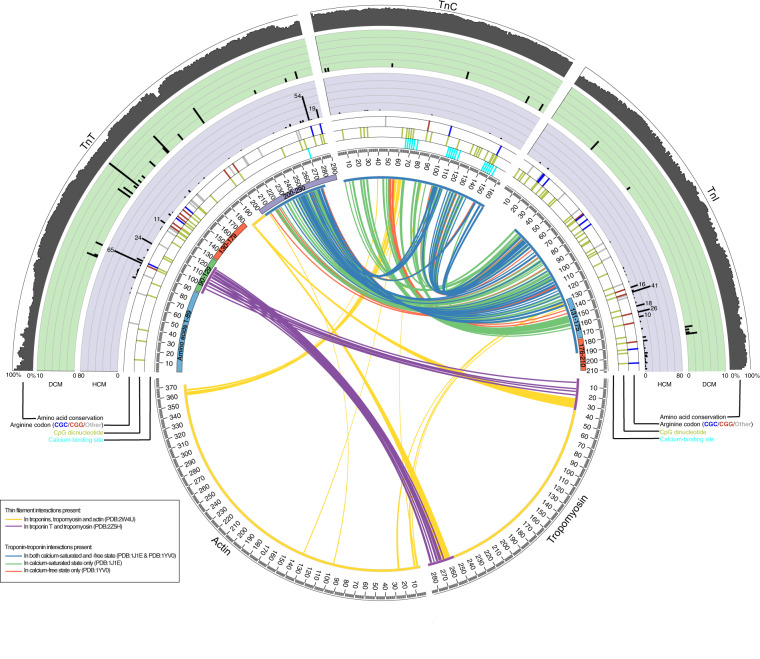
Human cardiac thin filament genetic variants and structural changes during calcium binding. The centre depicts interactions closer than 4 Å between pairs of residues in human cardiac thin filament are depicted as coloured arcs: interactions unique to calcium-saturated state (PDB:1J1E) (green); interactions unique to calcium-free state (PDB:1YV0) (red); interactions common to both calcium-saturated and calcium-free state (blue); interactions between troponins, tropomyosin and actin (PDB:2W4U) (yellow); and interactions between TnT and tropomyosin (PDB:2Z5H) (purple). Radiating out from the centre, rings show: extent of protein sequence resolved by crystallography; protein domains; amino acid residue numbers; location of calcium-binding residues (cyan); location and frequency of variants reported causative of hypertrophic cardiomyopathy (HCM) (purple background); location and frequency of variants reported causative of dilated cardiomyopathy (DCM) (green background); and conservation of each amino acid across ten species (grey histogram). Figure was generated by using Circos^11^.

We hypothesised the clinical outcome associated with variations in the Tn proteins may arise from their impact on the structure and dynamics of the protein complex. We analysed inter-subunit interactions in cardiac Tns and Tm in Ca^2+^ bound (Ca^2+^-saturated) and unbound (Ca^2+^-depleted) states (Fig. 1). The static amino acid interactions between subunits are independent of calcium binding, while dynamic interactions depend upon calcium binding (Fig. 1). Thus, we identified residues and regions among the Tns that have intermolecular interactions, and their calcium dependence (Supplementary Table 1). For TnT, these were residues 1–89, 90–129, 130–179 and 200–288; and for TnI, residues 131–175 and 176–210. These regions were later used for unbiased categorisation and analysis of clinical sub-classifications, therapy and patient outcomes.

The centre depicts interactions closer than 4 Å between pairs of residues in human cardiac thin filament are depicted as coloured arcs: interactions unique to calcium-saturated state (PDB:1J1E) (green); interactions unique to calcium-free state (PDB:1YV0) (red); interactions common to both calcium-saturated and calcium-free state (blue); interactions between Tns, Tm and actin (PDB:2W4U) (yellow); and interactions between TnT and Tm (PDB:2Z5H) (purple). Radiating out from the centre, rings show: extent of protein sequence resolved by crystallography; protein domains; amino acid residue numbers; location of calcium-binding residues (cyan); location and frequency of variants reported causative of HCM (purple background); location and frequency of variants reported causative of DCM (green background); and conservation of each amino acid across ten species (grey histogram). Figure was generated by using Circos^11^. Only the region 180–199, for which we did not have sufficient data to form part of our dynamic model was not used for future analysis of patient outcomes.

### Troponin variations tend to cluster to hotspots in regions that share similar clinical phenotypes

To understand how Tn structure correlates with genetic cardiomyopathies, we collected pathogenic amino acid substitutions reported in OMIM^12^ and ClinVar^13^ and identified cases from freely accessible publications through a systematic review. We identified 106 articles and collected 136 pathogenic or likely pathogenic amino acid substitutions in Tn genes: 13 in cardiac TnC (*TNNC1*), 65 in cardiac TnT (*TNNT2*) and 58 in cardiac TnI (*TNNI3*) (Supplemental Fig. 2). These variations were initially combined with the global case data from 981 patients (546 index cases and 435 relatives) from our systematic review. The full list of variations is reported in Supplementary Table 2. To study the distribution of variations in the Tns among patients, we plotted the frequencies of pathogenic substitutions associated with HCM and DCM using only the index cases along with intermolecular interactions (Fig. 1). We observed variations cluster and formed hotspots associated with either DCM or HCM in TnT and TnI. The localization of these variations may affect structural domains and their functions, e.g. residues involved in subunit interactions or calcium binding (Fig. 1 and Supplementary Table 1). Thus Fig. 1 synthesises information on clinical outcomes of genetic variations in patients with the structural and sequence details of the Tns and their dynamic interactions.

### Recurrent pathogenic variants occur at sites under negative selection

**Fig. 2:**
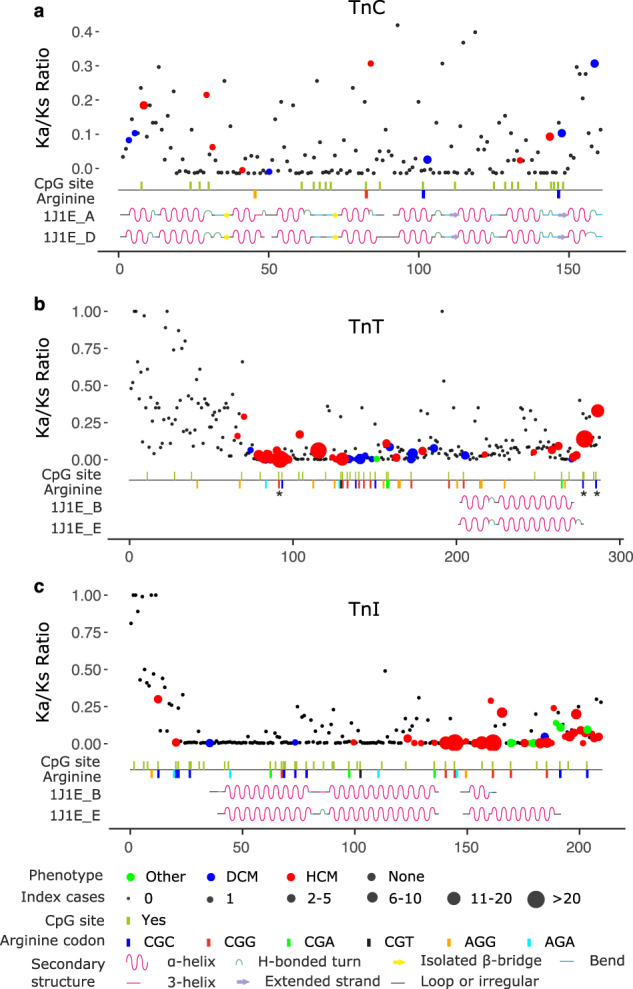
Summary of codon-specific selective pressure in human troponins. **a** Troponin C; **b** Troponin T; and **c** Troponin I. The amino acids sequences run on the *x*-axis and the selective pressure (represented by K_a_/K_s_ ratio) is on the *y*-axis. Each dot represents an amino acid in the protein sequences, the colour indicates disease phenotype and the size indicates the number of cases carrying variations at those sites. The CpG sites in the DNA sequence are marked by vertical bars. Codons for arginine are colour-coded. Secondary structure annotations are shown at the bottom. Asterisk indicates hotspot variation sites.

Evolutionarily conserved sites in protein sequences are crucial for maintaining protein structure and/or function; therefore, variations at these sites are largely deleterious. To investigate evolutionary conservation at sites of recurrent pathogenic variants, we analysed the conservation of amino acids in the TnC, TnT and TnI proteins by computing the site-specific rate of non-synonymous substitutions (K_a_) to the rate of synonymous substitutions (K_s_) at each site in orthologous sequences (K_a_/K_s_ ratio) . A K_a_/K_s_ ratio of 1.0 indicates neutral or no selection, smaller than 1.0 indicates a negative selection and greater than 1.0 indicates a positive selection. The ratio was calculated with programme Selecton and has been used previously e.g. in the highly reliable amino acid substitution pathogenicity predictor PON-P2^14,15^. Here, the K_a_/K_s_ ratios ranged from 1.6 × 10^−3^ to 1.0, with the majority of the amino acids having the ratio much less than 1.0. These results indicate that the protein sequences are highly conserved and under negative selection except in the N-termini (Fig. 2). Further the residues with recurrent variants in TnT and TnI have low Ka/Ks ratio, indicating the residues are under negative selection pressure.

### Arginine amino acids and CpG dinucleotides are mutation hotspots in troponin T

**Fig. 3:**
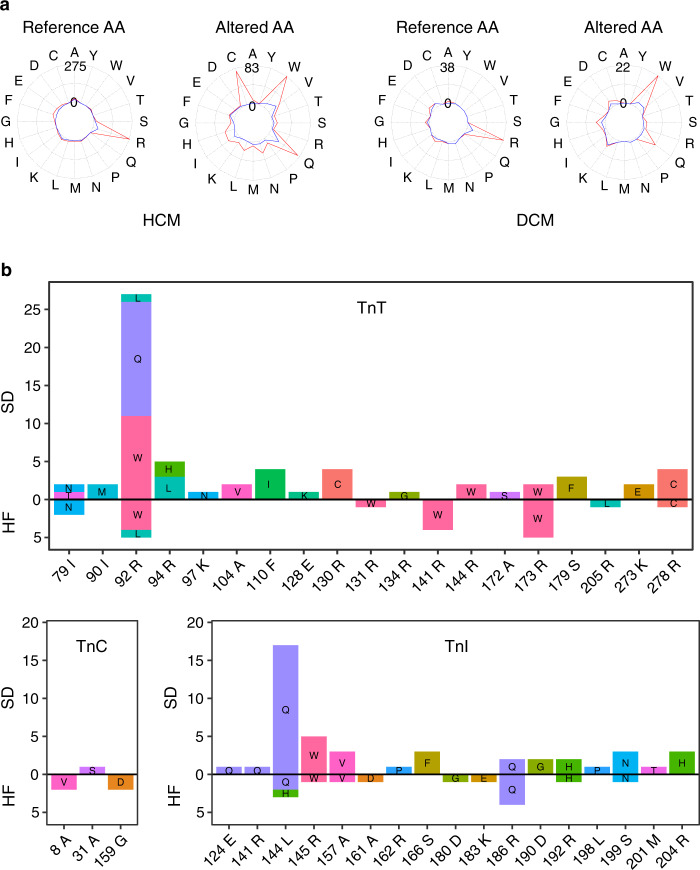
Distribution of amino acid variants for cause of death and phenotypes. **a** Frequencies of reference and altered amino acids in troponins among cases of HCM and DCM. The red line indicates total number of cases with either HCM or DCM and the blue line indicates cases carrying variants at known protein structure sites. Amino acid substitutions in troponins among index cases with mortality from heart failure (HF) or sudden cardiac death (SD). **b** Positions of protein sequence and reference amino acid are shown on *x*-axis and the mortality number on *y*-axis.

Arginine is the most frequently substituted amino acid in the Tns (Fig. 3). Changes to Cys, His, Glu and Trp are the most frequent in our data set (Supplementary Fig. 3a). As the numbers of index cases are the largest for TnT, we discuss cases in this protein. Of the index cases carrying a variant in TnT, 68.6% carried a variation of an arginine. Among the DCM patients with a variation in TnT, 78.7% (37 of 47 cases) had a variation of an arginine (Fig. 3a). Likewise, among HCM patients with a variation in TnT, 66.8% (167 of 250 cases) had a variation of an arginine (Fig. 3b). Arginine is coded by six codons: CGU, CGC, CGA, CGG, AGA and AGG. In the *TNNT2* gene, the most frequent codons for arginine are CGC (5 codons), CGG (9 codons) and AGG (12 codons). Codons CGC and CGG contain CpG dinucleotides. These dinucleotides are well-known variation hotspots in other genes^16^. Our results further support CpG dinucleotides are enriched as shown previously to TnT variants, but specific to this study we enhance this through our detailed analysis of HCM and DCM patients; Whilst also reporting limited anecdotal differences between HCM and DCM cases based on the underlying variation of the arginines in such regions. In particular, arginines in regions of TnT containing variations in HCM patients (i.e. amino acids 90–129 and 200–288) are often coded by CGC codons, whereas arginines in regions of TnT substituted in DCM patients (i.e. amino acids 130–179) are often coded by CGG codons (Figs. 1 and 2). Arg92 coded by CGG codon is an exception as 65 cases of HCM have been reported due to variations in this amino acid. However, it should be noted that given the limited size of the study our data did not show any statistical significance. Whilst, in the *TNNI3* gene, five of six sites containing a CGG codon had variations and we identified four of them as hotspots, whereas four of nine CGC codons contained variations, but were not hotspots. Thus, variations in HCM and DCM patients were frequently in arginines coded by CpG-containing codons.

### Simulations of hotspots of troponin T structure

**Fig. 4:**
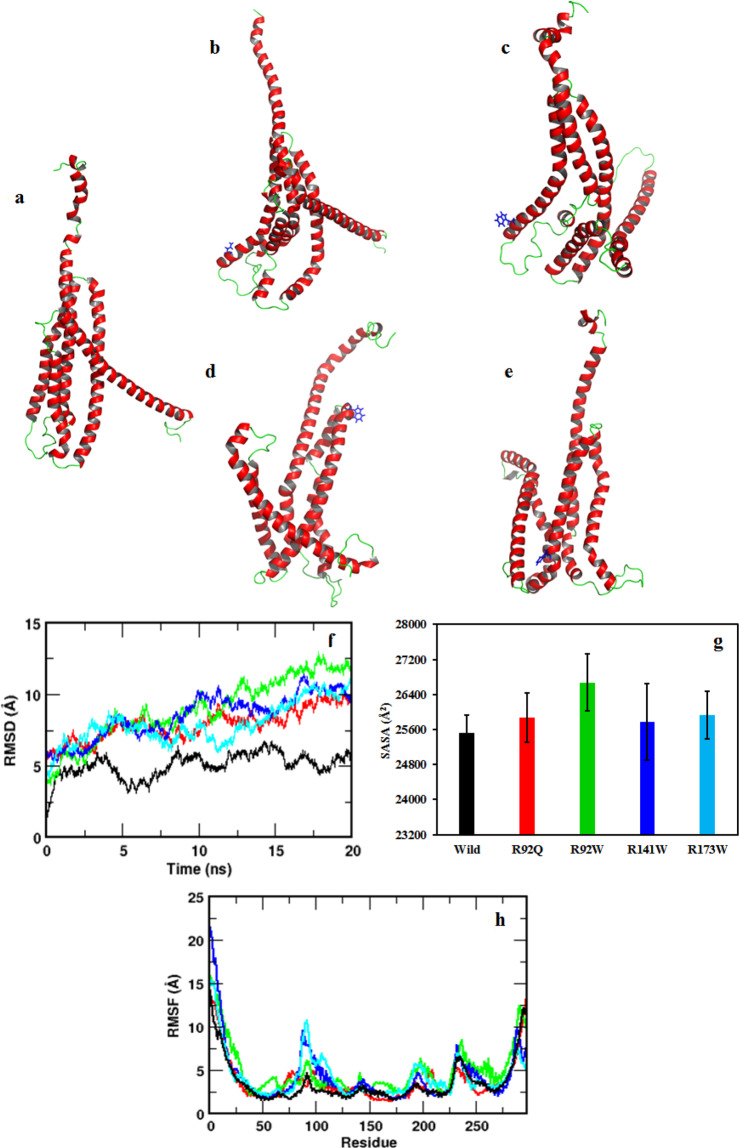
Molecular dynamics simulations of wild-type (WT) and TnT variants. Final conformations of TnT after 20 ns of simulation: **a** WT; **b** p.Arg92Gln; **c** p.Arg92Trp; **d** p.Arg141Trp and **e** p.Arg173Trp. **b**–**e** Location of amino acid variant is shown with blue sticks. **f**–**h** Structural analysis of simulated WT and variant TnTs: **f** Time evolution of backbone RMSDs of simulated TnTs from the equilibrated WT conformation. Structural deviations calculated in terms of RMSD values show that all the variants were experiencing larger conformational changes compared to the WT; **g** time-averaged accessible surface area for WT and variants over the simulation; and **h** ensemble-averaged root-mean-square fluctuations (RMSFs) of the C_α_ atoms in WT and variants, distinguishing the highly flexible regions in the protein. Colour scheme: WT (black), p.Arg92Gln (red), p.Arg92Trp (green), p.Arg141Trp (blue) and p.Arg173Trp (cyan).

To predict how variation hotspots pertaining to conformational changes may affect protein structure, we performed all-atom molecular dynamics simulations for wild-type and four common TnT variants. In the present study, simulation of the monomeric Tn unit was carried out to understand how the newly identified variations affect the structural integrity of the monomeric Tn rather than the complex formation and calcium sensitivity. The four variants underwent notable structural changes and deviated significantly from the wild-type structure after 20 ns of simulation, further the variant proteins lost the overall structural compactness during the course of simulations (Fig. 4b–e) (Supplementary Movies 1–5). The root-mean-square deviation (RMSD) values, computed by superposing each simulated snapshot onto the starting conformation, can provide insight into the degree of structural deviation experienced by the protein during the course of simulation. The wild type was structurally stabilized towards the end of simulation with a time-averaged RMSD of 5.0 ± 0.8 Å (Fig. 4f). Conversely, the variant forms underwent larger conformational changes, with the highest structural deviations in the p.Arg92Trp variant (Fig. 4f, green line). Moreover, in accordance with the RMSD values, the p.Arg92Trp variation was more solvent exposed than the others (Fig. 4g), suggesting the structural instability of the variants. The more dynamic nature of the variants was supported by their local fluctuations in the residues, as measured by the root-mean-square fluctuations (RMSFs) of their C_α_ atoms. Basically, RMSF calculates the degree of movement of each C_α_ atom around its average position, implying the highly flexible regions in the protein will show a large RMSF value while the more constrained regions will reflect a low RMSF. Residue fluctuations were increased in the variants (Supplementary Movies 1–5), with residues 80–125 being the most flexible (Fig. 4h). Most of the known TnT disease-related variations were clustered to the N-terminal end, which included the highly conserved region 112–136. Moreover, variations in the N-terminal region (e.g., p.Arg92Gln) weakening the folding and stability of the protein and complex formation with Tm are supported in previous studies^17,18^ (Supplementary Movies 1–5). Thus, the TnT variations perturbed the protein structure and its flexibility, which may subsequently lead to variation-specific cardiovascular phenotypes. However, detailed structural, biochemical and computational studies are required to explore the protein–protein interactions in the Tn complex and calcium-binding mechanism, which will be a focus of future research.

### Survival free of cardiovascular death does not differ between troponins, but clinical phenotypes and outcomes do vary

**Fig. 5:**
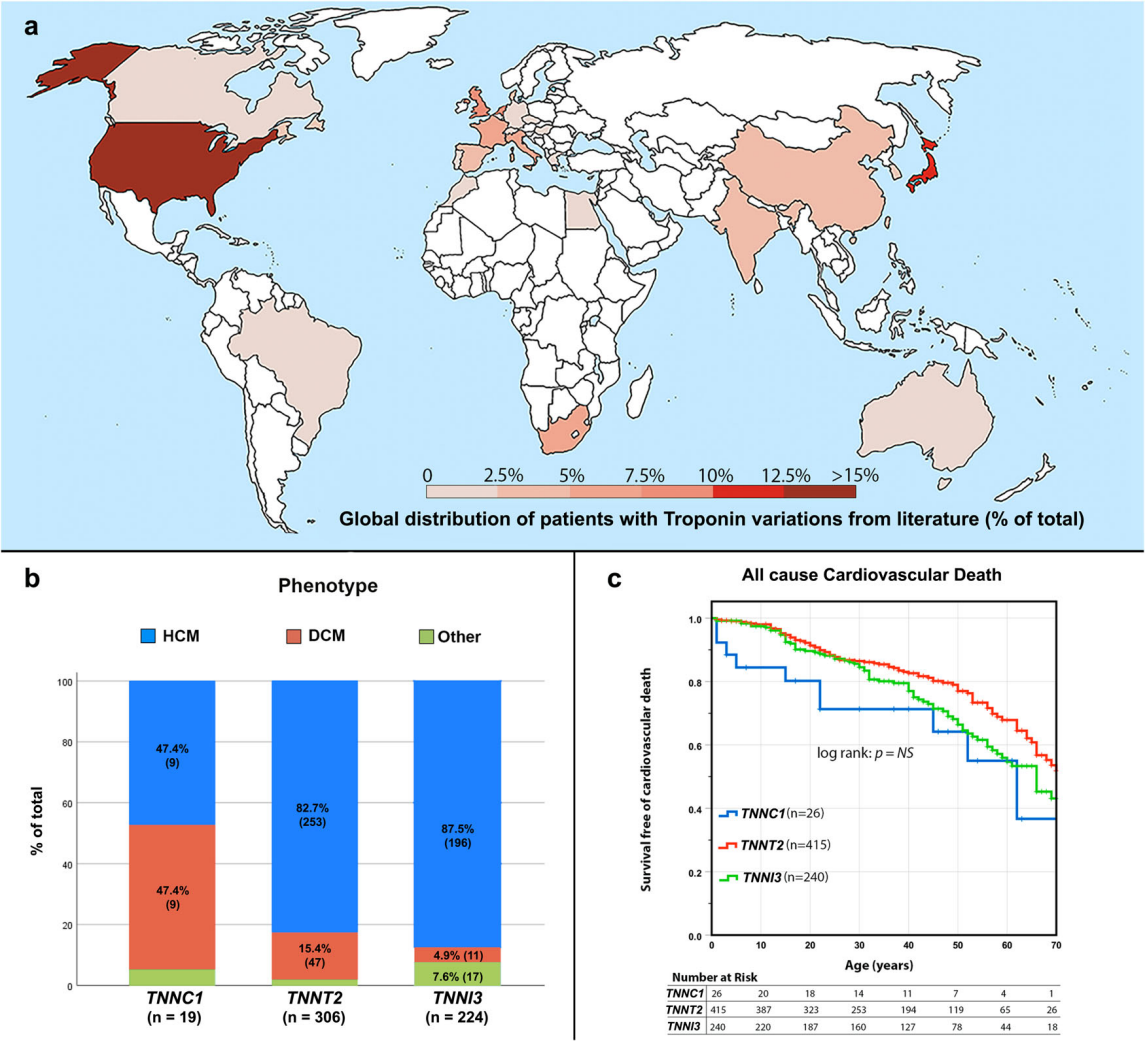
Variations in the cardiac troponins described in the literature. **a** Global distribution of cardiomyopathy patients across countries (% from total) carrying pathogenic or likely pathogenic substitutions in the troponin complex. **b** Associated phenotype according to the presence of substitutions in *TNNC1*, *TNNT2* and *TNNI3*. Differences were significant between the three groups (*p* < 0.001). **c** Survival curves showing the freedom from cardiovascular death in variations in *TNNC1*, *TNNT2* and *TNNI3* genes. The differences between genes did not reach statistical significance. *(The map was modified by us from this figure:https://commons.wikimedia.org/wiki/File:World_map_nations.svg. The figure has a Creative Commons licence so the figure is free to use and edit:* https://en.wikipedia.org/wiki/GNU_Free_Documentation_License*)*.

To identify global trends in contributions to cardiovascular death, we examined the clinical phenotypes and outcomes associated with variations in each patient’s Tn (Fig. 5a). Here we identified that HCM was the predominant phenotype in Tn variations: 83.4% of the probands reported in the literature had this phenotype (458/549). In *TNNT2*, HCM represented 82.7% (253/306) of the index cases with variations; in *TNNI3* the percentage was 87.5% (196/224) (Fig. 5b). The number of index cases reported with *TNNC1* variations was much smaller (only 19) than those with *TNNT2* and *TNNI3* variations; in this gene, the number of probands with DCM and HCM was balanced (47.4% for each phenotype; 9/19). For patients with variations in *TNNI3*, the proportion with DCM was lower than in the other two genes (only 4.9% developed this phenotype), but accounted for the highest proportion of patients with RCM (7.6%; 17/224). Thus, all three Tns had non-negligible association with different phenotypes (*p* < 0.001).

To understand the outcomes for patients with Tn variations, for 681 patients (including index and relatives) who had sufficient data to make a survival analysis at last follow-up age, we calculated the survival from global cardiovascular mortality and cardiovascular specific causes (SCD and heart failure death). However, the differences in cardiovascular mortality among the Tns did not reach statistical significance (Fig. 5c).

**Fig. 6:**
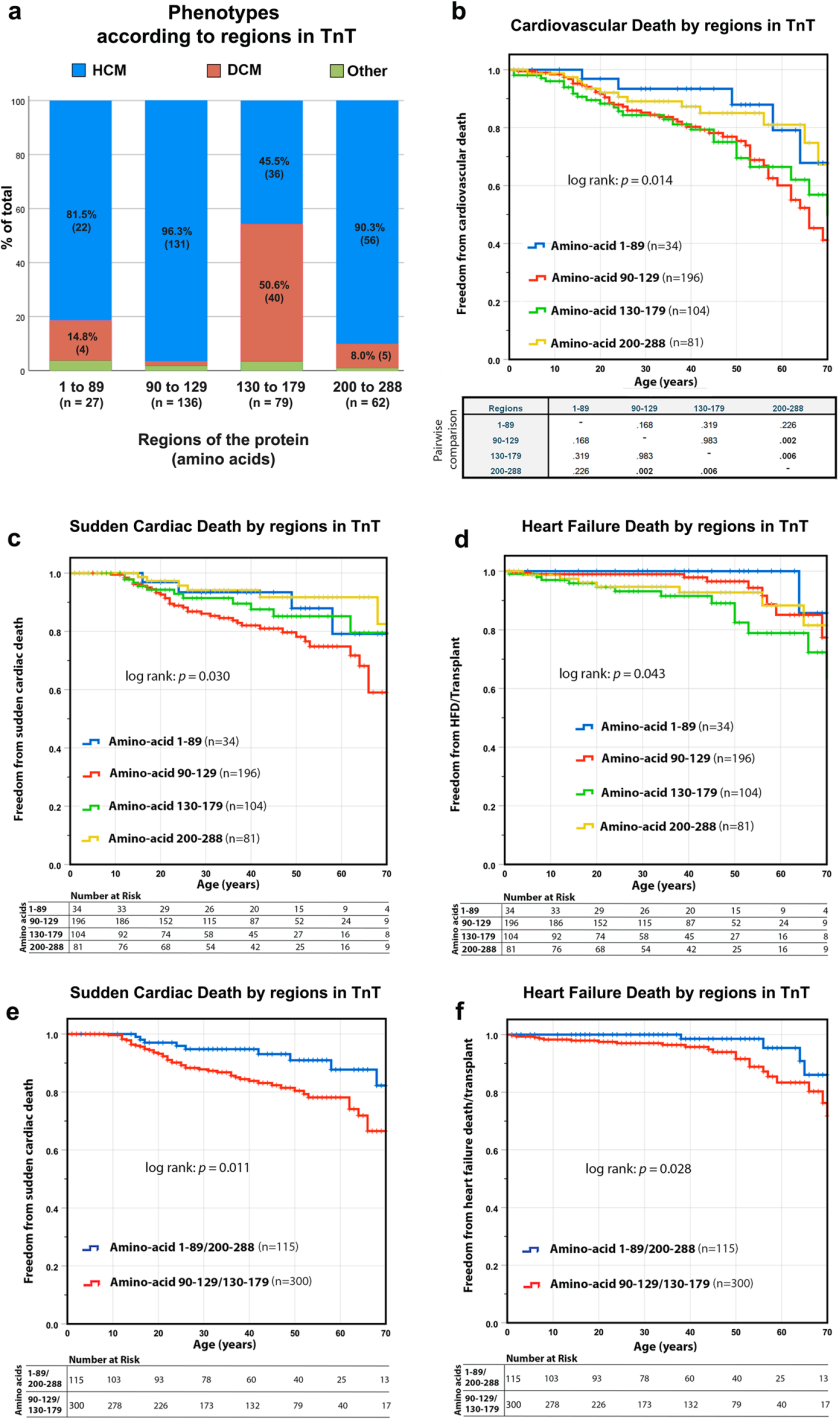
Clinical outcomes of amino acid substitutions in TnT regions. **a** Phenotypes according to regions in the TnT protein. HCM is significantly more frequent among patients with variations at amino acids 90–129 than in other regions; the higher number of DCM cases is observed in substitutions at amino acid 130–179 (*p* < 0.01). **b** Survival curve for the freedom from cardiovascular death in the four main regions of TnT; differences were statistically different between the groups (*p* = 0.014). Pairwise comparison showed a worse prognosis for patients with variations at regions 90–129 and 130–179 than at regions 1–89 and 200–288. **c** Freedom from sudden cardiac death in the different regions of TnT; differences were statistically significant (*p* = 0.030). Pairwise comparison showed a worse prognosis for individuals with variations in regions 90–129 and 130–179. **d** Freedom from heart failure death/transplant in the different regions of TnT; differences were statistically significant (*p* = 0.043). Pairwise comparison showed a worse prognosis for patients with variants in region 130–179 against regions 1–89, 90–129 and 130–179. **e**, **f** The same analysis performed by dividing TnT into high risk regions 90–129 and 130–179, and apparent lower risk regions 1–89 and 200–288. The difference is significant for sudden cardiac death (*p* = 0.011) and heart failure death/transplant (*p* = 0.028).

Variations in TnT showed separate hotspots for DCM and HCM (Fig. 1); thus, we examined the variable outcomes in these separate regions. More than 90% of the patients who had a variation among amino acids 90–129 or 200–288 had HCM; with amino acids 1–89 of TnT, HCM also predominated (81.5%) but the number of reported cases was lower than in the other regions (Fig. 6a). In contrast, DCM was the phenotype for 50.6% of patients with a variation among amino acids 130–179, with two sub-clusters: amino acids 130–150 and 172–173. Thus, patient phenotypes appeared correlated with the region in which their variation occurred. Further, there were differences in terms of cardiovascular death for variations in each region (*p* = 0.014) (Fig. 6b). Given TnT showed region-specific differences in the clinical outcome for variations, we repeated the survival analyses separately for each region (Fig. 6c–f). Variations among amino acids 90–129 and 130–179 were associated with the worst survival. When considering causes of cardiovascular death separately, there was a poor survival in terms of SCD for patients with a variation in amino acids 90–129 and 130–179 when compared to amino acids 1–89 and 200–288 (*p* = 0.030) (Fig. 6c). The worst prognosis in terms of heart failure death was for patients with a variation among amino acids 130–179 in comparison to any other region (*p* = 0.043) (Fig. 6d). The survival analysis was then performed by dividing TnT into regions of high risk (a variation among amino acids 90–129 and 130–180) and low risk (variations in amino acids 1–89 and 200–288). The difference between high- and low-risk regions was significant for SCD (*p* = 0.011) (Fig. 6e) and for heart failure death/transplant (*p* = 0.028) (Fig. 6f). Thus, there were significant differences in terms of cardiovascular death for variations in each region.

The variations in TnI highlighted two regions predominately associated with HCM, including amino acids 131–176, which interact with TnC in the calcium-bound state (Fig. 1). This may support the previous reports of HCM being a disease of increased calcium sensitivity and causing excessive calcium flux^18^. According to these data and the circos interactions we divided this gene in three different regions, involving amino acids 1–130, 131–175 and 176–210. There were non-statistically significant differences between the three regions in terms of survival of cardiovascular death (*p* = 0.084), probably because there were very few patients in region 1–130 (Supplementary Fig. 9). Pairwise comparison between regions 131–275 and 176–210 showed a worst survival for the latter (*p* = 0.008).

The variations in TnC were distributed along the length of the protein with no variation hotspots for HCM or DCM (Fig. 1). Further, the clinical phenotypes and prognosis attributable to variations were variable, and there were insufficient data from 26 patients to calculate survival curves.

## Discussion

In this study, we provide new perspectives for understanding correlations in the cardiomyopathies with Tn variations by developing a novel systems biology model that synthesises patient data on the prognosis and outcomes of Tn variations with structural data of the Tn complex. First, we independently determined relevant regions of the Tn proteins based on calcium-dependent interactions and sites under negative selection. Second, we identified variation hotspots and genotype-phenotype correlations and prognosis for each region by undertaking a metanalysis of the clinical data from freely accessible literature and public databases. Third, we modelled the likely deleterious effects of variations with molecular dynamics simulations with respect to the wild-type protein.

We like others have identified arginine codons and CpG dinucleotides as potential hotspots for cardiomyopathy-associated variations as well as other genetic disorders. Arginine is the most commonly substituted residue in many genetic diseases^19^, and its functions are only partly replaceable by other amino acids. Arginine is often functionally important due to its interactions with negatively charged residues to form salt bridges essential for protein stability. CpG sites are variation hotspots^20^ as cytosine is often methylated and can spontaneously deaminate to thymine, with the rate of transition from methylated cytosine to thymine 10- to 50-fold higher than for other nucleotides^16^. As four out of the six codons for arginine contain a CpG dinucleotide, their codons are particularly susceptible to variation.

From our data, we found arginine is the most frequently substituted amino acid amongst the Tns, and changes to Cys, His, Glu and Trp were the most frequent in our data set. Thus, particular arginine residues in the Tn family, e.g. p.Arg92 in TnT, are vulnerable to variation. Changes of residues at protein surface can be detrimental if the residues are involved in protein–ligand or protein–protein interactions. Thus, we surmise this to be relevant for Tns that form complexes with actin, Tm and Tn molecules, but requires further functional validation.

Our analyses identified TnT as possessing discrete regions within which variations were associated with similar prognoses and phenotypes for patients, likely because they cause a similar mechanistic impact. First, we identified two main regions of *TNNT2* that are rich in arginine codons and CpG dinucleotides. The first region codes for the Tm-binding region (residues 90–129), where HCM is the main phenotype for variations and includes well-known variations. For example, the p.Arg92Gln variation was one of the first described in the TnT and is associated with HCM^5,9^. The second region involves residues 130–179, in which variations associated with DCM are higher than in the other regions of the protein. Two thirds of the Tn variations associated with DCM occur in TnT, and the majority among residues 130–179 (Figs. 1 and 2). The gene for this zone is the richest in CGG codons. Although it is not a Tn interacting region, it is essential for the correct conformation of the protein, and variations altering its dynamic properties may lead to a defined patient phenotype. The prognosis in relation to cardiovascular death from our data is similar to the other regions, although heart failure, death/transplantation predominates over SCD as expected, these being the predominant phenotypes DCM and HCM, respectively (Fig. 6). In contrast, our data suggested variants in the third region, involving residues 200–288 in the C-terminal region of TnT, were mainly associated with HCM, and were clustered among amino acids 260–288, which binds to TnI. Compared to the rest of TnT, the 75 N-terminal amino acids are the least conserved across species, and the region is richer in acidic residues with fewer CpG dinucleotides. The most common variations, p.Arg278 and p. Arg286, are associated with HCM but with an incomplete penetrance and with a good prognosis, unless additional genetic or environmental modifiers are present^10^. Thus, variations in this region have a low incidence of cardiovascular events, hence we consider this part of the protein to be likely of low relevance for disease. Molecular dynamics simulations for four TnT variants in these regions predicted the structures of disease-related variants are more flexible than the wild type and display different conformations, which may impact their function (Fig. 4). Thus, our analyses suggest a future in which systems biology models combined with personalised clinical genomics can be used to understand how Tn variations in a cardiac patient may relate to their clinical phenotype, preferred treatment and likely outcome. Yet, we also appreciate that analysing clinical outcomes of variations is complicated by variable penetrance in patients carrying the same variation. These differences in disease progression may arise from the effects of differing environmental and lifestyle factors, as well as the contribution of individual variations in genetic backgrounds, modifier genes and epigenetic effects. However, accounting for these features will require extensive large-scale longitudinal clinical genomics studies. Nevertheless, we envisage integrative analyses, such as ours as enhancing methods for clinical risk stratification and to better define future clinical management decisions, especially within the inherited cardiac disease community. We hope the initiation of future multi-centre prospective trials will also facilitate integration and substantiate of such systems in real-world clinical practice.

Among some limitations of our study; clinical data were obtained after a systematic review that included all the evidence available in the literature, making the cohort of patients very heterogeneous. In addition, follow-up time was not summarized in all the papers, so conducting a sub-analysis of events since the diagnosis was not performed. On the same hand, combining data from probands and relatives could bias the analyses toward large families/founder variations.

This integrative systems biology study incorporates the largest independent and freely accessible systematic review on the inherited Tn cardiomyopathies. Although our data collection was rigorous, transparent and not confined to individual institutions, we were limited by the accessibility of published data and so greatly welcome the development of international multi-centre initiatives supporting data access. Our unbiased analysis based on the variations, and their impact on structural and physico-chemical interactions within the Tns of the thin filament complex provides insight into factors of variations for the development of differing phenotypes and clinical outcomes.

## Methods

### Sequence analysis

Protein sequences of the human cardiac thin filament proteins were taken from UniProt^21^ and included TnT (P45379), TnI (P19429), TnC (P63316), ACTC1 (P68032) and TPM1 (P09493). To cover a wide range of evolutionary history, orthologs of the human thin filament proteins were identified with BLASTP^22^ from the NCBI RefSeq database^23^. Sequences were selected from two mammals (human and mouse), two birds (chicken and ground tit (*Pseudopodoces humilis*)), two reptiles (Carolina anole (*Anolis carolinensis*) and Burmese python (*Python bivittatus*)), two amphibians (*Xenopus tropicalis* and *Xenopus laevis*) and two fishes (zebra fish (*Danio rerio*) and puffer fish (*Takifugu rubripes*)). The protein sequences of Tns were aligned with the orthologous human cardiac proteins by using MUSCLE^24^ followed by manual refinement in JalView^25^ (Supplementary Fig. 1). Conservation of each amino acid in the multiple sequence alignments for TnC, TnT and TnI was scored by using the Jensen–Shannon divergence^25^. Amino acid substitutions in TnC, TnT and TnI were taken from dbSNP^26^. Variants were classified according to dbSNP as: unknown; uncertain significance; (likely) benign; and (likely) pathogenic. If a variant was classified as both pathogenic and benign, then it was assigned a “disputed” status and excluded from the analysis.

### Protein structures and intermolecular interactions in the thin filament

To identify intermolecular residue–residue interactions between thin filament proteins, structures of complexes (Supplementary Table 3) were searched for pairs of residues with atoms within 4 Å radius for the human cardiac Tn in the calcium-bound state (PDB:1J1E)^27^, chicken skeletal muscle Tn in the calcium-free state (PDB:1YV0)^28^; a fragment of chicken skeletal muscle TnT bound to rabbit Tm (PDB:2Z5H)^29^; and an electron microscopy structure of the thin filament of insect flight muscle, into which were fitted the structure of chicken skeletal muscle Tn and rabbit skeletal muscle Tm and actin (PDB:2W4U)^30^. Corresponding residues were matched based on multiple sequence alignments. Residue–residue interactions were taken from the calcium-bound human cardiac Tn complex (PDB:1J1E); and mapped from chicken skeletal muscle TnT bound to rabbit Tm (PDB:2Z5H), and chicken skeletal muscle Tn and rabbit skeletal muscle Tm and actin (PDB:2W4U). As there is no structure for human calcium-free cardiac thin filament, residue–residue interactions were inferred from chicken skeletal muscle calcium-free Tn complex (PDB:1YV0); chicken skeletal muscle TnT bound to rabbit Tm (PDB:2Z5H), and chicken skeletal muscle Tn and rabbit skeletal muscle Tm and actin (PDB:2W4U). The residue–residue interactions of the human calcium-bound cardiac Tn complex (PDB:1J1E) and the chicken skeletal muscle calcium-free Tn complex (PDB:1YV0) were divided into three groups: (1) interactions unique to the human calcium-bound cardiac Tn complex; (2) interactions unique to the chicken skeletal muscle calcium-free Tn complex; and (3) interactions common to both the human calcium-bound cardiac Tn complex and the chicken skeletal muscle calcium-free Tn complex. When structural information was missing for the human proteins, we inferred interacting residues by mapping the residue–residue interactions in skeletal muscle Tns from other species.

### Literature search

We performed a comprehensive search of PubMed articles (1 January 1971 to 1 November 2019) to collect clinical information of families and individuals who carry amino acid substitutions in cardiac Tns associated with cardiomyopathy. The search terms used were:

“English” [Language] AND

(“1900/01/01” [Date-Publication]: “2019/11/31” [Date - Publication]) AND

("hypertrophic subaortic stenosis" OR

HSS* OR

"muscular subaortic stenosis" OR

"asymmetric septal hypertrophy" OR

ASH OR

"asymmetric septal hypertrophy" OR

"hypertrophic cardiomyopathy" OR

"hypertrophic obstructive cardiomyopathy" OR

"hypertrophic cardiomyopathies" OR

HCM OR

("dilated cardiomyopathy") OR

("restrictive cardiomyopathy" OR "restrictive cardiomyopathies")) AND

("troponin T type 2" OR "troponin T" OR TNNT2 OR “troponin C” OR TNNC1 OR "troponin I" OR TNNI3 OR troponins).

Titles and abstracts of the identified articles were evaluated by two experts. Next, the full article texts were evaluated and those meeting the following criteria were selected: observational English language reports describing phenotypic features (HCM; DCM; restrictive cardiomyopathy; left ventricular non-compaction) in patients with variations in genes *TNNC1*, *TNNT2* or *TNNI3*; and studies published in peer-reviewed journals. In addition, a manual search of the reference lists of the identified studies was performed, and references were evaluated using the same inclusion and exclusion criteria. Studies were included if they had information on relatives.

The collected information was stored into a database which includes for each study: country of origin (ISO country names); patient age at diagnosis, and last follow-up age; family history of cardiomyopathy; morphology and function of the heart evaluated by cardiac imaging (extent and pattern of hypertrophy, late gadolinium enhancement, atrial and ventricular dimensions and function, left ventricular outflow tract obstruction, mitral valve abnormalities); clinical risk factors for SCD (maximum left ventricular wall thickness ≥30 mm, abnormal exercise blood pressure response, non-sustained ventricular tachycardia, family history of SCD, syncope); interventions, outcome and prognosis (all death, cardiovascular death, SCD, non-fatal HF, AF, non-fatal stroke, implantable cardioverter-defibrillator implantation, myectomy, alcohol septal ablation).

All the variants were classified according to American Collage of Medical Genetics and Genomics^31,32^ guidelines for the interpretation of sequence variants. We considered the presence of the variant in control databases; number of studies and descriptive families; functional studies; evidence of co-segregation of the variant with the phenotype; and computational evidence support. A variant was considered pathogenic if it had causative variations in at least three independent peer-reviewed studies with non-contradictory evidence. If the evidence for pathogenicity was contradictory, internal information of more than 20,000 patients sequenced in Health In Code Database was used for support or reject pathogenicity for rare variants (i.e. case-control analysis was performed to define pathogenicity of variations in amino acids p.Arg278 and p.Arg286; Supplementary Table 4.). Every variant was reviewed by cardiologists specialized in genetics (L.M. and J.P.O.) who evaluated the evidence to confirm the variant classification. Only variants classified as pathogenic or likely pathogenic for cardiomyopathy were included.

For survival analyses, we defined the ‘cardiovascular death’ of a patient if one of the following was reported: (1) unexplained sudden death; (2) heart failure death or transplant; (3) stroke death; or (4) death related to a cardiovascular procedure (e.g. septal alcohol ablation). Patients were excluded from the analysis if they had complex phenotypes (i.e. more than one pathogenic or likely pathogenic variant in genes of the thin filament, or related to cardiomyopathy, e.g. *MYH7*, *MYBPC3* or *MYL2*). Survival analysis was made for 681 cases based on the latest follow-up. The cumulative probability for the occurrence of cardiovascular death was estimated with the Kaplan–Meier method and factors were compared by using the log-rank (Mantel–Cox) method. Survival was calculated from birth. A two-sided *p* value < 0.05 was considered statistically significant. Statistical analyses were performed by using IBM SPSS Statistics for Windows, Version 25.0 (Armonk, NY: IBM Corp).

### Visualisation of thin filament complexes

The Circos^11^ data visualisation tool was used to display and integrate information on proteins of thin filament complexes. Each of the proteins in the human complex is indicated as a segment in the circle. Arcs display intermolecular interactions between residues; histograms display residue conservation, and frequencies of variants in patients with DCM and HCM; and tiling displays locations of calcium-binding residues, and the location of amino acid substitutions reported in dbSNP^27^.

### Evolutionary conservation

The ratio of non-synonymous substitution rate to synonymous substitution rate (ω) estimates selective pressure. Synonymous variations are more common than non-synonymous variations, and thus ω is higher for variable sites than for conserved sites. Orthologous protein and cDNA sequences for Tns were collected from the Ensembl Compara database (http://www.ensembl.org/info/genome/compara/index.html) using its Perl application programming interface. Protein and cDNA sequences annotated as one-to-one orthologs were obtained. The numbers of protein and cDNA sequences analysed were 27 for TnC, 12 for TnI and 26 for TnT. The orthologous protein sequences were aligned using ClustalW^33^ and used for the codon alignment of cDNA sequences with PAL2NAL^34^. The cDNA codon alignment was provided to calculate codon-level ω^33^. The human sequence was used as the reference sequence.

### Homology modelling of human troponin T

Homology modelling of wild-type human TnT structure was performed with Robetta^35^, by using template structures and amino acid regions (Table 1). Out of the five models, we chose the best model based on its 3D quality assessed at the SAVES server^36^ and MolProbity^37^. The Ramachandran plots for the top five models were generated by using the RAMPAGE webserver (http://mordred.bioc.cam.ac.uk/~rapper/rampage.php).

**Table 1:** Template structures and amino acid sequences used to model human cardiac TnT.

Template PDB ID	Covered amino acids
4DLO chain E	1–81
2XS1 chain A	82–140
1XI4 chin J	141–195
1J1D chain C	196–298

### Molecular dynamics simulations

All-atom molecular dynamics simulations were performed for the model of wild-type TnT. The protonation states of residues were assigned based on the pK_a_ calculations at pH 7 by using the H++ server^38^. The protein was immersed in a cubic periodic box of TIP3P water model^39^ with water molecules extending 14 Å outside the protein atoms on all sides. The simulation box contained about 47,000 water molecules. Charge neutrality was maintained by adding 20 Na^+^ ions. Minimization and thermalization steps were performed by maintaining harmonic restraints on protein heavy atoms while the temperature was gradually raised to 300 K in canonical ensembles. The harmonic restraints were gradually reduced to zero and solvent density was adjusted under isobaric and isothermal conditions at 1 atm and 300 K. The system was equilibrated for 5 ns in NPT ensemble, with 2 fs simulation time steps. The equilibrated box dimensions were 120 Å × 160 Å × 80 Å. The energy components and system density converged. The system was further simulated to generate 20 ns of production data. The long-range electrostatic interactions were calculated by using Particle Mesh Ewald sum with a cutoff of 10 Å applied to Lennard–Jones interactions. The SHAKE algorithm^39^ was used to constrain all bonds involving hydrogen atoms. Variants were simulated in similar way. Four TnT variants—p.Arg92Gln, p.Arg92Trp, p.Arg141Trp and p.Arg173Trp—were generated by introducing a point variation in the equilibrated wild-type structure. The substitutions were performed with the Mutate Residue module in VMD^40^. The variant structures were equilibrated for 5 ns and simulated for 20 ns. The Amber 14.0 simulation software package with Amber ff99SB force field was used in all simulations^41^. Simulation trajectories were saved at intervals of 2 ps. Visualisations were done by using PyMOL^42^.

### Reporting summary

Further information on research design is available in the Nature Research Reporting Summary linked to this article.

## Supplementary information

Supplementary Information

Reporting Summary

Supplementary Movie titles

Supplementary Movie 1

Supplementary Movie 2

Supplementary Movie 3

Supplementary Movie 4

Supplementary Movie 5

## Data Availability

All data used for this study as part of our systematic review have been listed and accessible as listed on Supplementary Table 2. Clinical data sets are based on the data which was disclosed by the individual papers and trials listed.

## Code availability

We used R (v3.6.0), PAL2NAL (v14), selection (v2.3), ClustalW (v2.1) and Circos (v0.69-8). The custom codes used for generating figures are available on request to the authors.
