# Supplementary material for: Prognostic implications of troponin T variations in inherited cardiomyopathies using systems biology
Source: NPJ Genom Med. 2021 Jun 14;6:47. doi: 10.1038/s41525-021-00204-w (PMC8203786; doi:10.1038/s41525-021-00204-w)
Supplement: Supplementary file 1 — Supplementary Information [file 41525_2021_204_MOESM1_ESM.pdf]

**Supplementary Fig. 1 | Multiple sequence alignments of thin filament proteins.** Proteins are for cardiac isoforms, unless indicated otherwise. Sequences were aligned using MUSCLE, followed by manual refinement in JalView2. **a**, TnC; **b**, TnI and **c**, TnT. Residues: acidic (red); basic (blue); polar (green); aromatic (orange); aliphatic (pink); cysteine (yellow); and proline and glycine (magenta)

**a**

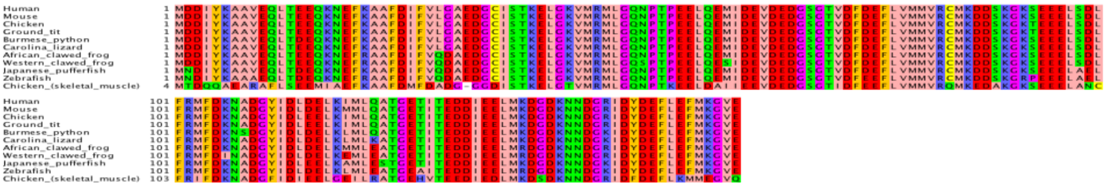

**b**

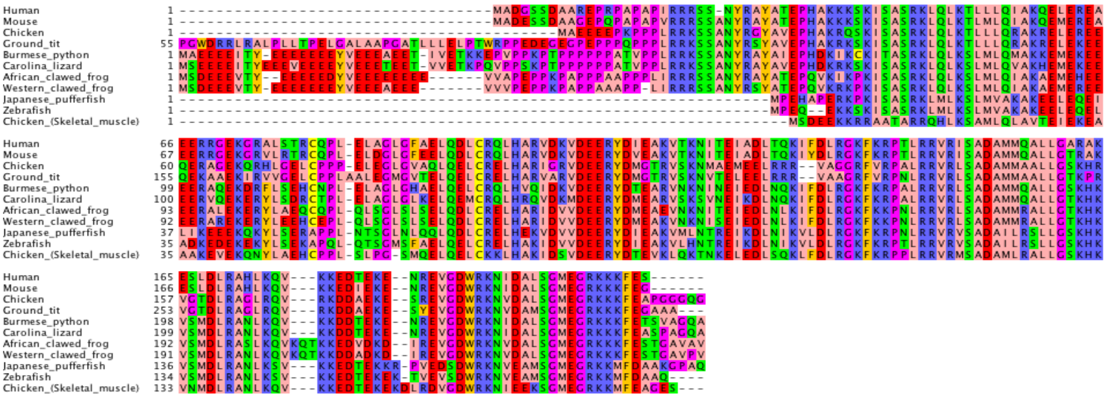

**c**

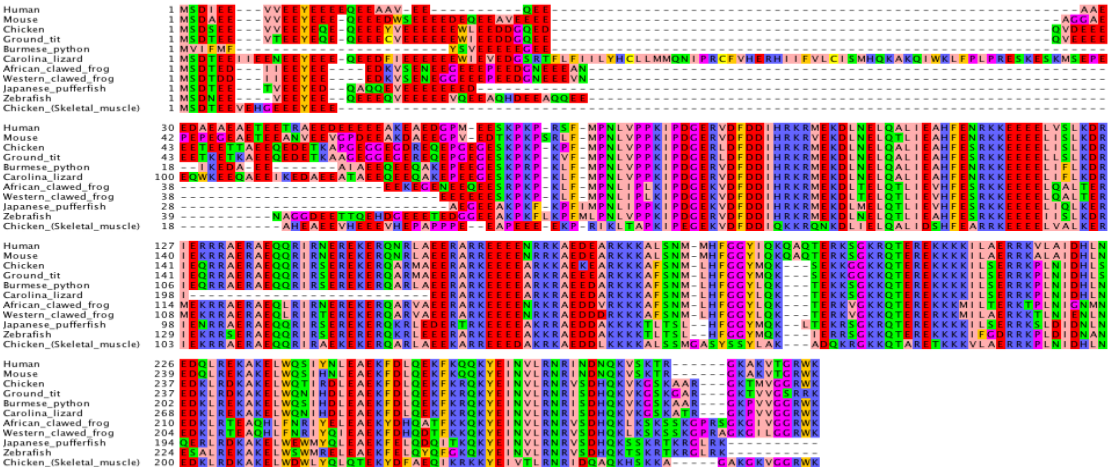

**Supplementary Fig. 2 | Flow chart of total number of studies used for meta-analysis of troponin variations in cardiomyopathies.**

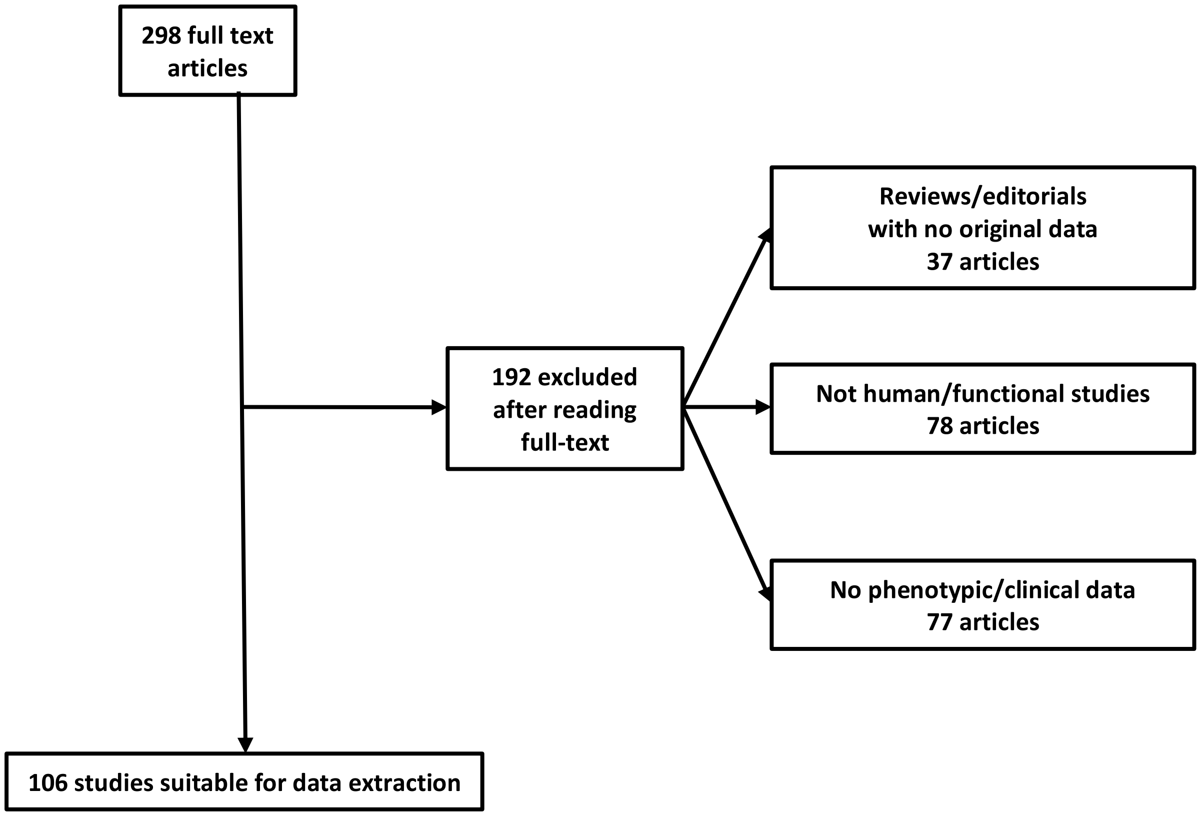

**Supplementary Fig.3 | Spider plot for the distributions of reference and altered amino acids among variants in troponins.** The red line indicates total number of cases, and the blue line indicates cases carrying variants at known protein structural regions.

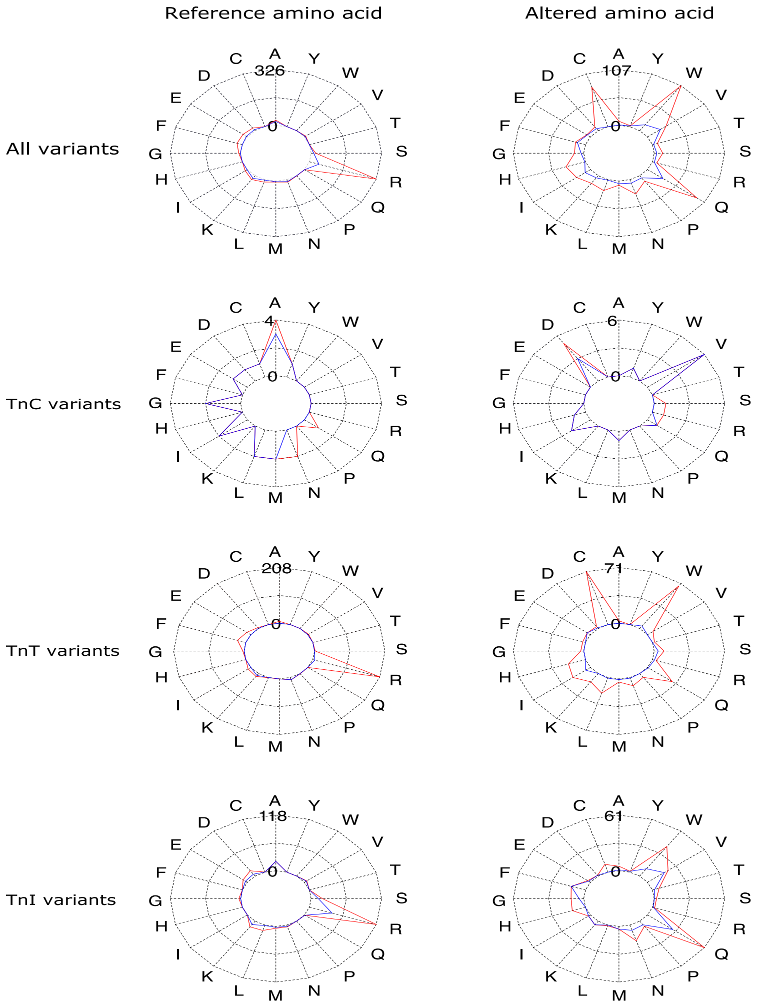

**Supplementary Table 1. Inter-molecular interactions observed between residues in structures of troponin complexes, and the pathology of associated hotspots. Individual interactions are shown in Fig. 1.**

| Protein | Region | Both $Ca^{2+}$ states | $Ca^{2+}$-saturated | $Ca^{2+}$-free | Tm | Actin | $Ca^{2+}$ cation | Hot spot |
|---|---|---|---|---|---|---|---|---|
| TnT | 1-89 | | | | | | | |
| | 90-129 | | | | + | | | HCM |
| | 130-179 | | | | | | | DCM |
| | 200-240 | | + | + | | | | |
| | 241-290 | + | + | | | | + | HCM |
| TnC | 1-100 | | + | + | | + | + | |
| | 101-160 | + | | | | | + | |
| TnI | 1-130 | + | + | + | | | | |
| | 131-175 | | + | + | | + | | HCM |
| | 176-210 | | | | | | | DCM |

**Supplementary Table 2**: List of mutations and references of all the articles finally included in the systematic review.

| | PROTEIN LEVEL | CHROMOSOMIC LEVEL | PAT. | Index (families) | Affected | RS | G.D. | SIFT | Polyphen-2 | Mutation Taster | References |
|---|---|---|---|---|---|---|---|---|---|---|---|
| ***TNNI3*** | NP_000354.4:p.Arg13Cys | NC_000019.9:g.55668489G>A | L.P. | 2 | 2 | | 180 | Damaging | Benign | | 1 |
| ***TNNI3*** | NP_000354.4:p.Arg21Cys | NC_000019.9:g.55668465G>A | L.P. | 3 | 4 | rs267607128 | 180 | Damaging | Probably damaging | Disease causing | 2, 3 |
| ***TNNI3*** | NP_000354.4:p.Lys36Gln | NC_000019.9:g.55668420T>G | P. | 3 | 5 | rs267607130 | 53 | Tolerated | Possibly damaging | Disease causing | 4, 5 |
| ***TNNI3*** | NP_000354.4:p.Arg74Pro | NC_000019.9:g.55667630C>G | L. P. | 1 | 1 | rs886039022 | 103 | Damaging | Probably damaging | Disease causing | 6 |
| ***TNNI3*** | NP_000354.4:p.Leu100Phe | NC_000019.9:g.55666183G>A | L.P. | 1 | 2 | rs773216333 | 22 | Damaging | Probably damaging | Disease causing | 7 |
| ***TNNI3*** | NP_000354.4:p.Glu124Gln | NC_000019.9:g.55666111C>G | LP. | 2 | 8 | rs727503506 | 29 | Damaging | Probably damaging | Disease causing | 8, 9 |
| ***TNNI3*** | NP_000354.4:p.Asp127Tyr | NC_000019.9:g.55665568C>A | LP | 1 | 1 | rs1114167340 | 160 | Damaging | Probably damaging | Disease causing | 10 |
| ***TNNI3*** | NP_000354.4:p.Gln130Arg | NC_000019.9:g.55665558T>C | LP. | 1 | 1 | rs1390435424 | 43 | Tolerated | Benign | Polymorphism | 11 |
| ***TNNI3*** | NP_000354.4:p.Arg136Gln | NC_000019.9:g.55665540C>T | L.P. | 1 | 1 | rs730881069 | 43 | Tolerated | Probably damaging | Disease causing | 12, 13 |
| ***TNNI3*** | NP_000354.4:p.Arg141Gln | NC_000019.9:g.55665525C>T | P. | 16 | 17 | rs397516347 | 43 | Damaging | Probably damaging | Polymorphism | 1, 14, 15, 16, 17, 18, 19, 20, 21, 22 |
| ***TNNI3*** | NP_000354.4:p.Leu144Gln | NC_000019.9:g.55665516A>T | P. | 4 | 31 | rs121917760 | 113 | Damaging | Probably damaging | Disease causing | 23, 24 |
| ***TNNI3*** | NP_000354.4:p.Leu144Pro | NC_000019.9:g.55665516A>G | L.P. | 1 | 1 | rs121917760 | 98 | Damaging | Probably damaging | Disease causing | 117 |
| ***TNNI3*** | NP_000354.4:p.Leu144His | NC_000019.9:g.55665515_55665516delCAinsAT | L.P. | 1 | 3 | | | | | | 116 |
| ***TNNI3*** | NP_000354.4:p.Arg145Gly | NC_000019.9:g.55665514G>C | P. | 3 | 11 | rs104894724 | 125 | Damaging | Probably damaging | Disease causing | 25, 26, 27 |
| ***TNNI3*** | NP_000354.4:p.Arg145Trp | NC_000019.9:g.55665514G>A | P. | 30 | 49 | rs104894724 | 101 | Damaging | Probably damaging | Disease causing | 7, 13, 17, 18, 21, 23, 28, 29, 30, 31 |
| ***TNNI3*** | NP_000354.4:p.Arg145Gln | NC_000019.9:g.55665513C>T | P. | 9 | 12 | rs397516349 | 43 | Damaging | Probably damaging | Disease causing | 7, 17, 18, 21, 26, 32 |
| ***TNNI3*** | NP_000354.4:p.Val147Leu | NC_000019.9:g.55665508C>G | LP. | 1 | 1 | rs777782551 | 32 | Damaging | Probably damaging | | 3 |
| ***TNNI3*** | NP_000354.4:p.Ser150Cys | NC_000019.9:g.55665498G>C | L.P. | 1 | 1 | | 112 | Damaging | Probably damaging | Disease causing | 13 |
| ***TNNI3*** | NP_000354.4:p.Met154Ile | NC_000019.9:g.55665485C>T | L.P. | 2 | 2 | rs397516350 | 10 | Damaging | Benign | Disease causing | 16 |
| ***TNNI3*** | NP_000354.4:p.Ala157Val | NC_000019.9:g.55665477G>A | P. | 18 | 23 | rs397516353 | 64 | Damaging | Possibly damaging | Disease causing | 16, 17, 18, 19, 21, 33 |
| ***TNNI3*** | NP_000354.4:p.Ala161Asp | NC_000019.9:g.55665465G>T | LP. | 1 | 1 | | 126 | Damaging | Benign | Disease causing | 34 |
| ***TNNI3*** | NP_000354.4:p.Arg162Trp | NC_000019.9:g.55665463G>A | P. | 8 | 8 | rs368861241 | 101 | Damaging | Probably damaging | Disease causing | 16, 26, 29, 35, 36 |
| ***TNNI3*** | NP_000354.4:p.Arg162Gln | NC_000019.9:g.55665462C>T | P. | 14 | 24 | rs397516354 | 43 | Tolerated | Possibly damaging | Disease causing | 15, 17, 18, 20, 36, 37, 38 |
| ***TNNI3*** | NP_000354.4:p.Arg162Pro | NC_000019.9:g.55665462C>G | P. | 4 | 6 | rs397516354 | 103 | Damaging | Possibly damaging | Disease causing | 19, 22, 37 |
| ***TNNI3*** | NP_000354.4:p.Lys164Thr | NC_000019.9:g.55665456T>G | L.P. | 1 | 1 | rs546367368 | 78 | Tolerated | Probably damaging | Disease causing | 21 |
| ***TNNI3*** | NP_000354.4:p.Ser166Phe | NC_000019.9:g.55665450G>A | P. | 12 | 16 | rs727504242 | 155 | Damaging | Possibly damaging | Disease causing | 17, 18, 20, 21, 29, 39, 40, 41 |
| ***TNNI3*** | NP_000354.4:p.Arg170Trp | NC_000019.9:g.55665439G>A | L.P. | 2 | 2 | rs727503504 | 101 | Damaging | Probably damaging | Disease causing | 7, 13 |
| ***TNNI3*** | NP_000354.4:p.Arg170Gln | NC_000019.9:g.55665438C>T | L.P. | 2 | 2 | rs727503503 | 43 | Damaging | Probably damaging | Disease causing | 42 |

| | PROTEIN LEVEL | CHROMOSOMIC LEVEL | PAT. | Index (families) | Affected | RS | G.D. | SIFT | Polyphen-2 | Mutation Taster | References |
|---|---|---|---|---|---|---|---|---|---|---|---|
| ***TNNI3*** | NP_000354.4:p.Ala171Thr | NC_000019.9:g.55665436C>T | L.P. | 1 | 1 | rs121917761 | 58 | Tolerated | Probably damaging | Disease causing | 23 |
| ***TNNI3*** | NP_000354.4:p.Val176Met | NC_000019.9:g.55665421C>T | LP. | 3 | 3 | rs727503501 | 21 | Damaging | Probably damaging | | 2, 22 |
| ***TNNI3*** | NP_000354.4:p.Lys178Glu | NC_000019.9:g.55665415T>C | L.P. | 1 | 1 | rs104894730 | 56 | Damaging | Probably damaging | Disease causing | 14, 23 |
| ***TNNI3*** | NP_000354.4:p.Asp180Gly | NC_000019.9:g.55665408T>C | L.P. | 4 | 7 | | 94 | Tolerated | Probably damaging | Disease causing | 5, 21, 43 |
| ***TNNI3*** | NP_000354.4:p.Glu182Lys | NC_000019.9:g.55665403C>T | L.P. | 1 | 1 | rs397516355 | 56 | Tolerated | Benign | Disease causing | 2 |
| ***TNNI3*** | NP_000354.4:p.Lys183Glu | NC_000019.9:g.55665400T>C | P. | 1 | 3 | rs730881077 | 56 | Tolerated | Benign | Disease causing | 17, 18 |
| ***TNNI3*** | NP_000354.4:p.Lys183Asn | NC_000019.9:g.55665398C>A | P. | 6 | 6 | | 94 | Damaging | Possibly damaging | Disease causing | 12, 13, 21 |
| ***TNNI3*** | NP_000354.4:p.Glu184Lys | NC_000019.9:g.55663285C>T | L.P. | 1 | 1 | rs397516356 | 56 | Tolerated | Benign | Disease causing | 44 |
| ***TNNI3*** | NP_000354.4:p.Asn185Lys | NC_000019.9:g.55663280G>T | L.P. | 3 | 4 | rs267607129 | 94 | Tolerated | Benign | Polymorphism | 4, 21, 45 |
| ***TNNI3*** | NP_000354.4:p.Arg186Gln | NC_000019.9:g.55663278C>T | P. | 9 | 26 | rs397516357 | 43 | Tolerated | Possibly damaging | Disease causing | 13, 17, 18, 19, 22, 46, 47 |
| ***TNII3*** | NP_000354.4:p.Val188Ala | NC_000019.9:g.55663272A>G | LP. | 1 | 2 | | 64 | Tolerated | Probably damaging | | 49 |
| ***TNNI3*** | NP_000354.4:p.Gly189Glu | NC_000019.9:g.55663269C>T | L. P. | 1 | 1 | rs587782980 | 98 | Tolerated | Probably damaging | Disease causing | 3 |
| ***TNNI3*** | NP_000354.4:p.Asp190Gly | NC_000019.9:g.55663266T>C | L.P. | 2 | 14 | rs104894728 | 94 | Damaging | Probably damaging | Disease causing | 23, 28 |
| ***TNNI3*** | NP_000354.4:p.Arg192Cys | NC_000019.9:g.55663261G>A | L.P. | 2 | 2 | rs727503499 | 180 | Damaging | Probably damaging | Disease causing | 13, 21 |
| ***TNNI3*** | NP_000354.4:p.Arg192His | NC_000019.9:g.55663260C>T | P. | 5 | 5 | rs104894729 | 29 | Damaging | Probably damaging | Disease causing | 23, 50, 51, 52 |
| ***TNNI3*** | NP_000354.4:p.Asn194Ser | NC_000019.9:g.55663254T>C | LP. | 1 | 1 | rs730881081 | 46 | Damaging | Probably damaging | Disease causing | 22 |
| ***TNNI3*** | NP_000354.4:p.Asp196Asn | NC_000019.9:g.55663249C>T | P. | 7 | 9 | rs104894727 | 23 | Damaging | Probably damaging | Disease causing | 3, 17, 18, 19, 22 |
| ***TNNI3*** | NP_000354.4:p.Leu198Val | NC_000019.9:g.55663243G>C | L.P. | 4 | 5 | rs727504285 | 32 | Tolerated | Possibly damaging | Disease causing | 222, 53 |
| ***TNNI3*** | NP_000354.4:p.Leu198Pro | NC_000019.9:g.55663242A>G | L.P. | 1 | 1 | | 98 | Tolerated | Probably damaging | Disease causing | 37 |
| ***TNNI3*** | NP_000354.4:p.Ser199Gly | NC_000019.9:g.55663240T>C | L.P. | 1 | 1 | | 56 | Damaging | Probably damaging | Disease causing | 17, 18 |
| ***TNNI3*** | NP_000354.4:p.Ser199Asn | NC_000019.9:g.55663239C>T | P. | 5 | 12 | rs730881091 | 46 | Damaging | Probably damaging | Disease causing | 17, 18, 30 |
| ***TNNI3*** | NP_000354.4:p.Met201Thr | NC_000019.9:g.55663233A>G | L.P. | 2 | 2 | rs727504365 | 81 | Damaging | Probably damaging | Disease causing | 21 |
| ***TNNI3*** | NP_000354.4:p.Glu202Gly | NC_000019.9:g.55663230T>C | L.P. | 1 | 1 | | 98 | Tolerated | Probably damaging | Disease causing | 17, 18 |
| ***TNNI3*** | NP_000354.4:p.Gly203Ser | NC_000019.9:g.55663228C>T | L.P. | 1 | 3 | rs267607127 | 56 | Damaging | Probably damaging | Disease causing | 26 |
| ***TNNI3*** | NP_000354.4:p.Gly203Arg | NC_000019.9:g.55663228C>G | L.P. | 1 | 2 | rs267607127 | 125 | Damaging | Probably damaging | Disease causing | 17, 18 |
| ***TNNI3*** | NP_000354.4:p.Arg204Cys | NC_000019.9:g.55663225G>A | L. P. | 1 | 1 | rs727504243 | 180 | Damaging | Probably damaging | Disease causing | 115 |
| ***TNNI3*** | NP_000354.4:p.Arg204His | NC_000019.9:g.55663224C>T | P. | 6 | 7 | rs727504275 | 29 | Damaging | Probably damaging | Disease causing | 37, 54, 55 |
| ***TNNI3*** | NP_000354.4:p.Lys206Gln | NC_000019.9:g.55663219T>G | L.P. | 1 | 1 | rs104894725 | 53 | Damaging | Probably damaging | Disease causing | 26 |
| ***TNNI3*** | NP_000354.4:p.Lys207Thr | NC_000019.9:g.55663215T>G | L.P. | 2 | 4 | rs3676722 | 78 | Damaging | Possibly damaging | Disease causing | 56 |
| ***TNNI3*** | NP_000354.4:p.Glu209Ala | NC_000019.9:g.55663209T>G | L.P. | 4 | 4 | rs730881083 | 107 | Damaging | Probably damaging | Disease causing | 21 |

| | PROTEIN LEVEL | CHROMOSOMIC LEVEL | PAT. | Index (families) | Affected | RS | G.D. | SIFT | Polyphen-2 | Mutation Taster | References |
|---|---|---|---|---|---|---|---|---|---|---|---|
| ***TNNC1*** | NP_003271.1:p.Asp3Val | NC_000003.11:g.52488024T>A | L.P. | 1 | 1 | rs730881063 | 152 | Damaging | Probably damaging | Disease causing | 113 |
| ***TNNC1*** | NP_003271.1:p.Tyr5His | NC_000003.11:g.52488019A>G | L.P. | 1 | 1 | | 83 | Tolerated | Possibly damaging | Disease causing | 5, 43, 57 |
| ***TNNC1*** | NP_003271.1:p.Ala8Val | NC_000003.11:g.52488009G>A | L.P. | 3 | 4 | rs267607125 | 64 | Tolerated | Possibly damaging | Disease causing | 58 |
| ***TNNC1*** | NP_003271.1:p.Leu29Gln | NC_000003.11:g.52486238A>T | L.P. | 1 | 1 | rs267607123 | 113 | Tolerated | Benign | Disease causing | 59 |
| ***TNNC1*** | NP_003271.1:p.Ala31Ser | NC_000003.11:g.52486233C>A | L.P. | 1 | 1 | rs397514616 | 99 | Damaging | Probably damaging | Disease causing | 60 |
| ***TNNC1*** | NP_003271.1:p.Leu41Met | NC_000003.11:g.52486203G>T | L. P. | 1 | 1 | | 15 | Damaging | Probably damaging | Disease causing | 114 |
| ***TNNC1*** | NP_003271.1:p.Gln50Arg | NC_000003.11:g.52486175T>C | P. | 1 | 4 | | 43 | Damaging | Possibly damaging | Disease causing | 5, 61 |
| ***TNNC1*** | NP_003271.1:p.Cys84Tyr | NC_000003.11:g.52485826C>T | L.P. | 1 | 1 | rs267607126 | 194 | Damaging | Probably damaging | Disease causing | 58 |
| ***TNNC1*** | NP_003271.1:p.Met103Ile | NC_000003.11:g.52485768C>G | L.P. | 2 | 5 | | 10 | Tolerated | Benign | Disease causing | 43, 57 |
| ***TNNC1*** | NP_003271.1:p.Glu134Asp | NC_000003.11:g.52485459C>A | L.P. | 1 | 1 | rs397516847 | 45 | Tolerated | Possibly damaging | Disease causing | 58 |
| ***TNNC1*** | NP_003271.1:p.Asn144Asp | NC_000003.11:g.52485431T>C | L. P. | 2 | 5 | rs730881061 | 23 | Damaging | Benign | Disease causing | 7, 112 |
| ***TNNC1*** | NP_003271.1:p.Ile148Val | NC_000003.11:g.52485419T>C | L.P. | 2 | 2 | rs397516848 | 29 | Tolerated | Benign | Disease causing | 43, 57, 63 |
| ***TNNC1*** | NP_003271.1:p.Gly159Asp | NC_000003.11:g.52485301C>T | P. | 2 | 9 | rs104893823 | 94 | Damaging | Probably damaging | Disease causing | 5, 29, 64 |
| ***TNNT2*** | NP_001001430.1:p.Lys66Gln | NC_000001.10:g.201335973T>G | L. P. | 1 | 1 | | 53 | Damaging | Probably damaging | Disease causing | 22 |
| ***TNNT2*** | NP_001001430.1:p.Phe70Leu | NC_000001.10:g.201334794A>G | L.P. | 1 | 1 | | 22 | Tolerated | Probably damaging | Disease causing | 19 |
| ***TNNT2*** | NP_001001430.1:p.Leu74Phe | NC_000001.10:g.201334780C>A | L.P. | 1 | 1 | | 22 | Damaging | Probably damaging | Disease causing | 92 |
| ***TNNT2*** | NP_001001430.1:p.Ile79Asn | NC_000001.10:g.201334766A>T | P. | 7 | 18 | rs121964855 | 194 | Damaging | Probably damaging | Disease causing | 65, 66, 67, 68 |
| ***TNNT2*** | NP_001001430.1:p.Ile79Thr | NC_000001.10:g.201334766A>G | L.P. | 1 | 1 | rs121964855 | 89 | Damaging | Probably damaging | Disease causing | 69 |
| ***TNNT2*** | NP_001001430.1:p.Pro80Ser | NC_000001.10:g.201334764G>A | L. P. | 1 | 2 | rs397516451 | 74 | Damaging | Probably damaging | Disease causing | 53 |
| ***TNNT2*** | NP_001001430.1:p.Gly82Arg | NC_000001.10:g.201334758C>T | L.P. | 2 | 4 | rs727504255 | 125 | Damaging | Probably damaging | Disease causing | 70 |
| ***TNNT2*** | NP_001001430.1:p.Glu83Lys | NC_000001.10:g.201334755C>T | L.P. | 2 | 4 | rs727504244 | 56 | Damaging | Probably damaging | Disease causing | 66 |
| ***TNNT2*** | NP_001001430.1:p.Glu83Asp | NC_000001.10:g.201334753C>G | L.P. | 2 | 2 | rs727503514 | 45 | Tolerated | Benign | Disease causing | 44, 71 |
| ***TNNT2*** | NP_001001430.1:p.Arg84Thr | NC_000001.10:g.201334751C>G | L.P. | 1 | 1 | rs397516452 | 71 | Damaging | Probably damaging | Disease causing | 108 |
| ***TNNT2*** | NP_001001430.1:p.Arg84Ser | NC_000001.10:g.201334750T>A | L.P. | 1 | 1 | rs397516453 | 110 | Damaging | Probably damaging | Disease causing | 107 |
| ***TNNT2*** | NP_001001430.1:p.Val85Met | NC_000001.10:g.201334749C>T | L.P. | 1 | 2 | | 21 | Damaging | Probably damaging | Disease causing | 72 |
| ***TNNT2*** | NP_001001430.1:p.Val85Leu | NC_000001.10:g.201334749C>G | L.P. | 2 | 2 | | 32 | Damaging | Benign | Disease causing | 73 |
| ***TNNT2*** | NP_001001430.1:p.Asp86Ala | NC_000001.10:g.201334745T>G | L.P. | 3 | 3 | rs397516455 | 126 | Damaging | Probably damaging | Disease causing | 20 |
| ***TNNT2*** | NP_001001430.1:p.Phe87Leu | NC_000001.10:g.201334743A>G | P. | 1 | 11 | | 22 | Tolerated | Probably damaging | Disease causing | 74 |
| ***TNNT2*** | NP_001001430.1:p.Ile90Met | NC_000001.10:g.201334430G>C | P. | 2 | 15 | | 10 | Damaging | Probably damaging | Disease causing | 75 |
| ***TNNT2*** | NP_001001430.1:p.Arg92Trp | NC_000001.10:g.201334426G>A | P. | 41 | 84 | rs397516456 | 101 | Damaging | Probably damaging | Disease causing | 20, 26, 53, 66, 69, 73, 76, 77, 78, 79, 80, 81, 82 |

| | PROTEIN LEVEL | CHROMOSOMIC LEVEL | PAT. | Index (families) | Affected | RS | G.D. | SIFT | Polyphen-2 | Mutation Taster | References |
|---|---|---|---|---|---|---|---|---|---|---|---|
| ***TNNT2*** | NP_001001430.1:p.Arg92Gln | NC_000001.10:g.201334425C>T | P. | 23 | 64 | rs121964856 | 43 | Tolerated | Probably damaging | Disease causing | 65, 66, 67, 76, 78, 83, 84, 85, 86 |
| ***TNNT2*** | NP_001001430.1:p.Arg92Leu | NC_000001.10:g.201334425C>A | P. | 4 | 8 | rs121964856 | 102 | Damaging | Probably damaging | Disease causing | 19, 66 |
| ***TNNT2*** | NP_001001430.1:p.Arg94Cys | NC_000001.10:g.201334420G>A | P. | 4 | 4 | rs727503513 | 180 | Damaging | Probably damaging | Disease causing | 53, 73 |
| ***TNNT2*** | NP_001001430.1:p.Arg94His | NC_000001.10:g.201334419C>T | L.P. | 5 | 5 | rs397516457 | 29 | Damaging | Probably damaging | Disease causing | 13, 72 |
| ***TNNT2*** | NP_001001430.1:p.Arg94Leu | NC_000001.10:g.201334419C>A | P. | 5 | 7 | rs397516457 | 102 | Damaging | Probably damaging | Disease causing | 26, 56, 66, 87 |
| ***TNNT2*** | NP_001001430.1:p.Glu96Lys | NC_000001.10:g.201334414C>T | L.P. | 1 | 4 | | 56 | Damaging | Probably damaging | Disease causing | 88 |
| ***TNNT2*** | NP_001001430.1:p.Lys97Asn | NC_000001.10:g.201334409C>A | L.P. | 2 | 3 | rs397516459 | 94 | Damaging | Probably damaging | Disease causing | 89 |
| ***TNNT2*** | NP_001001430.1:p.Ala104Val | NC_000001.10:g.201334389G>A | L.P. | 3 | 6 | rs727504245 | 64 | Tolerated | Possibly damaging | Polymorphism | 63, 66 |
| ***TNNT2*** | NP_001001430.1:p.Phe110Ile | NC_000001.10:g.201334372A>T | P. | 16 | 31 | rs121964858 | 21 | Damaging | Probably damaging | Disease causing | 26, 53, 65, 69, 73, 76, 90 |
| ***TNNT2*** | NP_001001430.1:p.Phe110Leu | NC_000001.10:g.201334372A>G | P. | 6 | 8 | rs121964858 | 22 | Damaging | Probably damaging | Disease causing | 76, 84 |
| ***TNNT2*** | NP_001001430.1:p.Phe110Val | NC_000001.10:g.201334372A>C | L.P. | 2 | 7 | rs121964858 | 50 | Damaging | Probably damaging | Disease causing | 19, 91 |
| ***TNNT2*** | NP_001001430.1:p.Lys124Asn | NC_000001.10:g.201334328T>A | L.P. | 1 | 1 | | 94 | Damaging | Probably damaging | Disease causing | 92 |
| ***TNNT2*** | NP_001001430.1:p.Arg126Trp | NC_000001.10:g.201334324T>A | L.P. | 1 | 1 | rs786204405 | 101 | Damaging | Probably damaging | Disease causing | 16 |
| ***TNNT2*** | NP_001001430.1:p.Glu128Lys | NC_000001.10:g.201333503C>T | L.P. | 5 | 5 | rs730881100 | 56 | Damaging | Probably damaging | Disease causing | 1, 41 |
| ***TNNT2*** | NP_001001430.1:p.Arg130Cys | NC_000001.10:g.201333497G>A | P. | 11 | 16 | rs397516463 | 180 | Damaging | Probably damaging | Disease causing | 26, 69, 76, 84, 92 |
| ***TNNT2*** | NP_001001430.1:p.Arg131Trp | NC_000001.10:g.201333494G>A | P. | 5 | 6 | rs74315380 | 101 | Damaging | Probably damaging | Disease causing | 29, 44, 93, 94 |
| ***TNNT2*** | NP_001001430.1:p.Arg134Gly | NC_000001.10:g.201333485G>C | P. | 3 | 11 | rs45525839 | 125 | Damaging | Probably damaging | Disease causing | 5, 44, 94, 95 |
| ***TNNT2*** | NP_001001430.1:p.Arg139His | NC_000001.10:g.201333469C>T | L.P. | 1 | 1 | rs397516466 | 29 | Damaging | Probably damaging | Disease causing | 94 |
| ***TNNT2*** | NP_001001430.1:p.Arg141Trp | NC_000001.10:g.201333464G>A | P. | 11 | 30 | rs74315379 | 101 | Damaging | Probably damaging | Disease causing | 5, 14, 44, 94, 96 |
| ***TNNT2*** | NP_001001430.1:p.Arg144Trp | NC_000001.10:g.201333455G>A | L.P. | 1 | 6 | rs483352832 | 101 | Damaging | Probably damaging | Disease causing | 97 |
| ***TNNT2*** | NP_001001430.1:p.Arg148Trp | NC_000001.10:g.201333443G>A | L. P. | 1 | 1 | rs730881123 | 101 | Damaging | Probably damaging | Disease causing | 118 |
| ***TNNT2*** | NP_001001430.1:p.Arg151Cys | NC_000001.10:g.201333434G>A | L.P. | 1 | 1 | rs45608937 | 180 | Damaging | Probably damaging | Disease causing | 5, 94, 95 |
| ***TNNT2*** | NP_001001430.1:p.Ala157Ser | NC_000001.10:g.201332525C>A | L.P. | 2 | 2 | | 99 | Tolerated | Possibly damaging | Disease causing | 12, 13 |
| ***TNNT2*** | NP_001001430.1:p.Arg159Gln | NC_000001.10:g.201332518C>T | L.P. | 2 | 2 | rs45501500 | 43 | Tolerated | Probably damaging | Disease causing | 5, 94, 95, 98 |
| ***TNNT2*** | NP_001001430.1:p.Glu163Lys | NC_000001.10:g.201332507C>T | L.P. | 5 | 8 | | 56 | Tolerated | Possibly damaging | Disease causing | 25, 53, 65, 66 |
| ***TNNT2*** | NP_001001430.1:p.Ala172Ser | NC_000001.10:g.201332480C>A | L.P. | 2 | 8 | rs730881097 | 99 | Tolerated | Probably damaging | Disease causing | 81, 99 |
| ***TNNT2*** | NP_001001430.1:p.Arg173Trp | NC_000001.10:g.201332477G>A | P. | 4 | 25 | rs727503512 | 101 | Damaging | Probably damaging | Disease causing | 13, 100 |
| ***TNNT2*** | NP_001001430.1:p.Arg173Gln | NC_000001.10:g.201332476C>T | P. | 3 | 9 | rs397516471 | 43 | Damaging | Probably damaging | Disease causing | 44., 101 |
| ***TNNT2*** | NP_001001430.1:p.Ser179Phe | NC_000001.10:g.201332458G>A | L.P. | 2 | 6 | rs727504246 | 155 | Damaging | Probably damaging | | 102 |

| | PROTEIN LEVEL | CHROMOSOMIC LEVEL | PAT. | Index (families) | Affected | RS | G.D. | SIFT | Polyphen-2 | Mutation Taster | References |
|---|---|---|---|---|---|---|---|---|---|---|---|
| ***TNNT2*** | NP_001001430.1:p.Gly186Val | NC_000001.10:g.201332437C>A | L.P. | 2 | 2 | | 109 | Damaging | Probably damaging | Disease causing | 103 |
| ***TNNT2*** | NP_001001430.1:p.Arg205Trp | NC_000001.10:g.201331117G>A | L.P. | 4 | 4 | rs45586240 | 101 | Damaging | Probably damaging | Disease causing | 5, 45, 95 |
| ***TNNT2*** | NP_001001430.1:p.Arg205Gln | NC_000001.10:g.201331116C>T | L. P. | 1 | 4 | rs121964860 | 43 | Damaging | Probably damaging | Disease causing | 119 |
| ***TNNT2*** | NP_001001430.1:p.Arg205Leu | NC_000001.10:g.201331116C>A | L.P. | 1 | 3 | rs121964860 | 102 | Damaging | Probably damaging | Disease causing | 5, 29, 94 |
| ***TNNT2*** | NP_001001430.1:p.Lys217Met | NC_000001.10:g.201331080T>A | L. P. | 1 | 1 | | 95 | Damaging | Probably damaging | Disease causing | 2 |
| ***TNNT2*** | NP_001001430.1:p.Lys247Arg | NC_000001.10:g.201330447T>C | L. P. | 2 | 2 | | 26 | Tolerated | Probably damaging | Disease causing | 83, 111 |
| ***TNNT2*** | NP_001001430.1:p.Lys258Ile | NC_000001.10:g.201330414T>A | L. P. | 2 | 2 | rs397516482 | 102 | Damaging | Probably damaging | Disease causing | 107, 108 |
| ***TNNT2*** | NP_001001430.1:p.Asn262Asp | NC_000001.10:g.201328788T>C | L.P. | 1 | 1 | | 23 | Damaging | Probably damaging | Disease causing | 78 |
| ***TNNT2*** | NP_001001430.1:p.Asn262Ser | NC_000001.10:g.201328787T>C | L.P. | 4 | 4 | rs397516483 | 23 | Tolerated | Probably damaging | Disease causing | 22, 109, 110 |
| ***TNNT2*** | NP_001001430.1:p.Asp270Asn | NC_000001.10:g.201328764C>T | L.P. | 1 | 2 | rs121964861 | 23 | Damaging | Probably damaging | Disease causing | 29, 94 |
| ***TNNT2*** | NP_001001430.1:p.Asn271Ile | NC_000001.10:g.201328760T>A | P. | 4 | 6 | | 149 | Tolerated | Probably damaging | Disease causing | 16, 19 |
| ***TNNT2*** | NP_001001430.1:p.Lys273Glu | NC_000001.10:g.201328755T>C | P. | 4 | 12 | | 56 | Damaging | Probably damaging | Disease causing | 53, 73, 85 |
| ***TNNT2*** | NP_001001430.1:p.Arg278Cys | NC_000001.10:g.201328373G>A | P. | 49 | 65 | rs121964857 | 180 | Damaging | Probably damaging | Disease causing | 12, 13, 20, 38, 42, 65, 66, 76, 83, 84, 86, 104, 105 |
| ***TNNT2*** | NP_001001430.1:p.Arg278His | NC_000001.10:g.201328372C>T | L.P. | 1 | 1 | rs397516484 | 29 | Tolerated | Probably damaging | Disease causing | 66 |
| ***TNNT2*** | NP_001001430.1:p.Arg278Pro | NC_000001.10:g.201328372C>G | P. | 4 | 6 | rs397516484 | 103 | Tolerated | Probably damaging | Disease causing | 20, 41, 104 |
| ***TNNT2*** | NP_001001430.1:p.Arg278Leu | NC_000001.10:g.201328372C>A | L.P. | 1 | 1 | | 102 | Tolerated | Probably damaging | Disease causing | 78 |
| ***TNNT2*** | NP_001001430.1:p.Arg286Cys | NC_000001.10:g.201328349G>A | P. | 7 | 9 | rs367785431 | 180 | Damaging | Probably damaging | Disease causing | 19, 104, 106 |
| ***TNNT2*** | NP_001001430.1:p.Arg286His | NC_000001.10:g.201328348C>T | L.P. | 10 | 12 | rs141121678 | 29 | Damaging | Probably damaging | Disease causing | 20, 30 |
| ***TNNT2*** | NP_001001430.1:p.Arg286Pro | NC_000001.10:g.201328348C>G | L.P. | 2 | 2 | | 103 | Damaging | Probably damaging | Disease causing | 3 |

**PAT:** pathogenicity; **LP**: likely pathogenic; **P:** pathogenic; **Affec:** affected carriers; **G.D:** Grahntam's distance (conservation).

**References from Supplemental Table 2:**


1. Zou Y, Wang J, Liu X, Wang Y, Chen Y, Sun K, et al. Multiple gene mutations, not the type of mutation, are the modifier of left ventricle hypertrophy in patients with hypertrophic cardiomyopathy. Mol Biol Rep [Internet]. 2013 Jun;40(6):3969–76. Available from: http://www.ncbi.nlm.nih.gov/pubmed/23283745
2. Arad M, Penas-Lado M, Monserrat L, Maron BJ, Sherrid M, Ho CY, et al. Gene mutations in apical hypertrophic cardiomyopathy. Circulation [Internet]. 2005 Nov 1;112(18):2805–11. Available from: http://www.ncbi.nlm.nih.gov/pubmed/16267253
3. Berge KE, Leren TP. Genetics of hypertrophic cardiomyopathy in Norway. Clin Genet. 2014;86(4):355–60.
4. Carballo S, Robinson P, Otway R, Fatkin D, Jongbloed JDH, de Jonge N, et al. Identification and functional characterization of cardiac troponin I as a novel disease gene in autosomal dominant dilated cardiomyopathy. Circ Res [Internet]. 2009 Aug 14;105(4):375–82. Available from: http://www.ncbi.nlm.nih.gov/pubmed/19590045
5. Rampersaud E, Siegfried JD, Norton N, Li D, Martin E, Hershberger RE. Rare variant mutations identified in pediatric patients with dilated cardiomyopathy. Prog Pediatr Cardiol [Internet]. 2011 Jan 1;31(1):39–47. Available from: http://www.ncbi.nlm.nih.gov/pubmed/21483645
6. Boda U, Vadapalli S, Calambur N, Nallari P. Novel mutations in beta-myosin heavy chain, actin and troponin-I genes associated with dilated cardiomyopathy in Indian population. J Genet [Internet]. 2009 Dec;88(3):373–7. Available from: http://www.ncbi.nlm.nih.gov/pubmed/20086309
7. Robyns T, Kuiperi C, Breckpot J, Devriendt K, Souche E, Van Cleemput J, et al. Repeat genetic testing with targeted capture sequencing in primary arrhythmia syndrome and cardiomyopathy. Eur J Hum Genet [Internet]. 2017;25(12):1313–23. Available from: http://www.ncbi.nlm.nih.gov/pubmed/29255176
8. Das K J, Ingles J, Bagnall RD, Semsarian C. Determining pathogenicity of genetic variants in hypertrophic cardiomyopathy: importance of periodic reassessment. Genet Med [Internet]. 2014 Apr;16(4):286–93. Available from: http://www.ncbi.nlm.nih.gov/pubmed/24113344
9. Chiou K-R, Chu C-T, Charng M-J. Detection of mutations in symptomatic patients with hypertrophic cardiomyopathy in Taiwan. J Cardiol [Internet]. 2015 Mar;65(3):250–6. Available from: http://www.ncbi.nlm.nih.gov/pubmed/25086479
10. Sheng J-J, Jin J-P. Gene regulation, alternative splicing, and posttranslational modification of troponin subunits in cardiac development and adaptation: a focused review. Front Physiol [Internet]. 2014;5:165. Available from: http://www.ncbi.nlm.nih.gov/pubmed/24817852
11. Morita H, Larson MG, Barr SC, Vasan RS, O'Donnell CJ, Hirschhorn JN, et al. Single-gene mutations and increased left ventricular wall thickness in the community: the Framingham Heart Study. Circulation [Internet]. 2006 Jun 13;113(23):2697–705. Available from: http://www.ncbi.nlm.nih.gov/pubmed/16754800
12. Teirlinck CH, Senni F, Malti R El, Majoor-Krakauer D, Fellmann F, Millat G, et al. A human MYBPC3 mutation appearing about 10 centuries ago results in a hypertrophic cardiomyopathy with delayed onset, moderate evolution but with a risk of sudden death. BMC Med Genet [Internet]. 2012 Nov 10;13:105. Available from: http://www.ncbi.nlm.nih.gov/pubmed/23140321
13. Millat G, Bouvagnet P, Chevalier P, Dauphin C, Jouk PS, Da Costa A, et al. Prevalence and spectrum of mutations in a cohort of 192 unrelated patients with hypertrophic cardiomyopathy. Eur J Med Genet [Internet]. 53(5):261–7. Available from: http://www.ncbi.nlm.nih.gov/pubmed/20624503

14. Kaski JP, Syrris P, Burch M, Tomé-Esteban M-T, Fenton M, Christiansen M, et al. Idiopathic restrictive cardiomyopathy in children is caused by mutations in cardiac sarcomere protein genes. Heart [Internet]. 2008 Nov;94(11):1478–84. Available from: http://www.ncbi.nlm.nih.gov/pubmed/18467357
15. Rani DS, Nallari P, Priyamvada S, Narasimhan C, Singh L, Thangaraj K. High prevalence of Arginine to Glutamine substitution at 98, 141 and 162 positions in Troponin I (TNNI3) associated with hypertrophic cardiomyopathy among Indians. BMC Med Genet [Internet]. 2012 Aug 10;13:69. Available from: http://www.ncbi.nlm.nih.gov/pubmed/22876777
16. Santos S, Marques V, Pires M, Silveira L, Oliveira H, Lança V, et al. High resolution melting: improvements in the genetic diagnosis of hypertrophic cardiomyopathy in a Portuguese cohort. BMC Med Genet [Internet]. 2012 Mar 19;13:17. Available from: http://www.ncbi.nlm.nih.gov/pubmed/22429680
17. Moon JC, Mogensen J, Elliott PM, Smith GC, Elkington AG, Prasad SK, et al. Myocardial late gadolinium enhancement cardiovascular magnetic resonance in hypertrophic cardiomyopathy caused by mutations in troponin I. Heart [Internet]. 2005 Aug;91(8):1036–40. Available from: http://www.ncbi.nlm.nih.gov/pubmed/16020591
18. Mogensen J, Murphy RT, Kubo T, Bahl A, Moon JC, Klausen IC, et al. Frequency and clinical expression of cardiac troponin I mutations in 748 consecutive families with hypertrophic cardiomyopathy. J Am Coll Cardiol [Internet]. 2004 Dec 21;44(12):2315–25. Available from: http://www.ncbi.nlm.nih.gov/pubmed/15607392
19. Richard P, Charron P, Carrier L, Ledeuil C, Cheav T, Pichereau C, et al. Hypertrophic cardiomyopathy: distribution of disease genes, spectrum of mutations, and implications for a molecular diagnosis strategy. Circulation [Internet]. 2003 May 6;107(17):2227–32. Available from: http://www.ncbi.nlm.nih.gov/pubmed/12707239
20. Van Driest SL, Ellsworth EG, Ommen SR, Tajik AJ, Gersh BJ, Ackerman MJ. Prevalence and spectrum of thin filament mutations in an outpatient referral population with hypertrophic cardiomyopathy. Circulation [Internet]. 2003 Jul 29;108(4):445–51. Available from: http://www.ncbi.nlm.nih.gov/pubmed/12860912
21. van den Wijngaard A, Volders P, van Tintelen JP, Jongbloed JDH, van den Berg MP, Lekanne Deprez RH, et al. Recurrent and founder mutations in the Netherlands: Cardiac troponin I (TNNI3) gene mutations as a cause of severe forms of hypertrophic and restrictive cardiomyopathy. Netherlands Hear J. 2011;19(8):344–51.
22. Coppini R, Ho CY, Ashley E, Day S, Ferrantini C, Girolami F, et al. Clinical phenotype and outcome of hypertrophic cardiomyopathy associated with thin-filament gene mutations. J Am Coll Cardiol [Internet]. 2014 Dec 23;64(24):2589–600. Available from: http://www.ncbi.nlm.nih.gov/pubmed/25524337
23. Mogensen J, Kubo T, Duque M, Uribe W, Shaw A, Murphy R, et al. Idiopathic restrictive cardiomyopathy is part of the clinical expression of cardiac troponin I mutations. J Clin Invest [Internet]. 2003 Jan;111(2):209–16. Available from: http://www.ncbi.nlm.nih.gov/pubmed/12531876
24. Arbustini E, Narula N, Tavazzi L, Serio A, Grasso M, Favalli V, et al. The MOGE(S) classification of cardiomyopathy for clinicians. J Am Coll Cardiol. 2014;64(3):304–18.
25. Choi J-O, Yu C-W, Chun Nah J, Rang Park J, Lee B-S, Jeong Choi Y, et al. Long-term outcome of 4 Korean families with hypertrophic cardiomyopathy caused by 4 different mutations. Clin Cardiol [Internet]. 2010 Jul;33(7):430–8. Available from: http://www.ncbi.nlm.nih.gov/pubmed/20641121
26. Kimura A, Harada H, Park JE, Nishi H, Satoh M, Takahashi M, et al. Mutations in the cardiac troponin I gene associated with hypertrophic cardiomyopathy. Nat Genet [Internet]. 1997 Aug;16(4):379–82. Available from: http://www.ncbi.nlm.nih.gov/pubmed/9241277

27. Zhao Y, Feng Y, Zhang Y-M, Ding X-X, Song Y-Z, Zhang A-M, et al. Targeted Next-Generation Sequencing Reveals Hot Spots and Doubly Heterozygous Mutations in Chinese Patients with Familial Cardiomyopathy. Biomed Res Int [Internet]. 2015;2015:561819. Available from: http://www.ncbi.nlm.nih.gov/pubmed/26199943
28. Kubo T, Gimeno JR, Bahl A, Steffensen U, Steffensen M, Osman E, et al. Prevalence, clinical significance, and genetic basis of hypertrophic cardiomyopathy with restrictive phenotype. J Am Coll Cardiol [Internet]. 2007 Jun 26;49(25):2419–26. Available from: http://www.ncbi.nlm.nih.gov/pubmed/17599605
29. Mogensen J, Murphy RT, Shaw T, Bahl A, Redwood C, Watkins H, et al. Severe disease expression of cardiac troponin C and T mutations in patients with idiopathic dilated cardiomyopathy. J Am Coll Cardiol [Internet]. 2004 Nov 16;44(10):2033–40. Available from: http://www.ncbi.nlm.nih.gov/pubmed/15542288
30. Andersen PS, Havndrup O, Hougs L, Sørensen KM, Jensen M, Larsen LA, et al. Diagnostic yield, interpretation, and clinical utility of mutation screening of sarcomere encoding genes in Danish hypertrophic cardiomyopathy patients and relatives. Hum Mutat [Internet]. 2009 Mar;30(3):363–70. Available from: http://www.ncbi.nlm.nih.gov/pubmed/19035361
31. Hwang J-W, Jang M-A, Jang SY, Seo SH, Seong M-W, Park SS, et al. Diverse Phenotypic Expression of Cardiomyopathies in a Family with TNNI3 p.Arg145Trp Mutation. Korean Circ J [Internet]. 2017 Mar;47(2):270–7. Available from: http://www.ncbi.nlm.nih.gov/pubmed/28382084
32. Wang J, Wang Y, Zou Y, Sun K, Wang Z, Ding H, et al. Malignant effects of multiple rare variants in sarcomere genes on the prognosis of patients with hypertrophic cardiomyopathy. Eur J Heart Fail [Internet]. 2014 Sep;16(9):950–7. Available from: http://www.ncbi.nlm.nih.gov/pubmed/25132132
33. Weissler-Snir A, Hindieh W, Gruner C, Fourey D, Appelbaum E, Rowin E, et al. Lack of Phenotypic Differences by Cardiovascular Magnetic Resonance Imaging in MYH7 (β-Myosin Heavy Chain)- Versus MYBPC3 (Myosin-Binding Protein C)-Related Hypertrophic Cardiomyopathy. Circ Cardiovasc Imaging [Internet]. 2017 Feb;10(2). Available from: http://www.ncbi.nlm.nih.gov/pubmed/28193612
34. Kawai H, Morimoto S-I, Takakuwa Y, Ueda A, Inada K-I, Sarai M, et al. Hypertrophic Cardiomyopathy Accompanied by Spinocerebellar Atrophy With a Novel Mutation in Troponin I Gene. Int Heart J [Internet]. 2016 Jul 27;57(4):507–10. Available from: http://www.ncbi.nlm.nih.gov/pubmed/27385602
35. Garcia-Pavia P, Vázquez ME, Segovia J, Salas C, Avellana P, Gómez-Bueno M, et al. Genetic basis of end-stage hypertrophic cardiomyopathy. Eur J Heart Fail [Internet]. 2011 Nov;13(11):1193–201. Available from: http://www.ncbi.nlm.nih.gov/pubmed/21896538
36. Alejandra Restrepo-Cordoba M, Campuzano O, Ripoll-Vera T, Cobo-Marcos M, Mademont-Soler I, Gámez JM, et al. Usefulness of Genetic Testing in Hypertrophic Cardiomyopathy: an Analysis Using Real-World Data. J Cardiovasc Transl Res [Internet]. 2017 Feb;10(1):35–46. Available from: http://www.ncbi.nlm.nih.gov/pubmed/28138913
37. Doolan A, Tebo M, Ingles J, Nguyen L, Tsoutsman T, Lam L, et al. Cardiac troponin I mutations in Australian families with hypertrophic cardiomyopathy: clinical, genetic and functional consequences. J Mol Cell Cardiol [Internet]. 2005 Feb;38(2):387–93. Available from: http://www.ncbi.nlm.nih.gov/pubmed/15698845
38. Gruner C, Care M, Siminovitch K, Moravsky G, Wigle ED, Woo A, et al. Sarcomere protein gene mutations in patients with apical hypertrophic cardiomyopathy. Circ Cardiovasc Genet [Internet]. 2011 Jun;4(3):288–95. Available from: http://www.ncbi.nlm.nih.gov/pubmed/21511876

39. Santori M, Blanco-Verea A, Gil R, Cortis J, Becker K, Schneider PM, et al. Broad-based molecular autopsy: a potential tool to investigate the involvement of subtle cardiac conditions in sudden unexpected death in infancy and early childhood. Arch Dis Child [Internet]. 2015 Oct;100(10):952–6. Available from: http://www.ncbi.nlm.nih.gov/pubmed/26272908
40. Brion M, Allegue C, Santori M, Gil R, Blanco-Verea A, Haas C, et al. Sarcomeric gene mutations in sudden infant death syndrome (SIDS). Forensic Sci Int [Internet]. 2012 Jun 10;219(1–3):278–81. Available from: http://www.ncbi.nlm.nih.gov/pubmed/22361390
41. Erdmann J, Daehmlow S, Wischke S, Senyuva M, Werner U, Raible J, et al. Mutation spectrum in a large cohort of unrelated consecutive patients with hypertrophic cardiomyopathy. Clin Genet [Internet]. 2003 Oct;64(4):339–49. Available from: http://www.ncbi.nlm.nih.gov/pubmed/12974739
42. Kaski JP, Syrris P, Esteban MTT, Jenkins S, Pantazis A, Deanfield JE, et al. Prevalence of sarcomere protein gene mutations in preadolescent children with hypertrophic cardiomyopathy. Circ Cardiovasc Genet [Internet]. 2009 Oct;2(5):436–41. Available from: http://www.ncbi.nlm.nih.gov/pubmed/20031618
43. Hershberger RE, Norton N, Morales A, Li D, Siegfried JD, Gonzalez-Quintana J. Coding sequence rare variants identified in MYBPC3, MYH6, TPM1, TNNC1, and TNNI3 from 312 patients with familial or idiopathic dilated cardiomyopathy. Circ Cardiovasc Genet [Internet]. 2010 Apr;3(2):155–61. Available from: http://www.ncbi.nlm.nih.gov/pubmed/20215591
44. Lakdawala NK, Funke BH, Baxter S, Cirino AL, Roberts AE, Judge DP, et al. Genetic testing for dilated cardiomyopathy in clinical practice. J Card Fail [Internet]. 2012 Apr;18(4):296–303. Available from: http://www.ncbi.nlm.nih.gov/pubmed/22464770
45. van Spaendonck-Zwarts KY, van Rijsingen IAW, van den Berg MP, Lekanne Deprez RH, Post JG, van Mil AM, et al. Genetic analysis in 418 index patients with idiopathic dilated cardiomyopathy: overview of 10 years' experience. Eur J Heart Fail [Internet]. 2013 Jun;15(6):628–36. Available from: http://www.ncbi.nlm.nih.gov/pubmed/23349452
46. Roberts WC, Roberts CC, Ko JM, Grayburn PA, Tandon A, Kuiper JJ, et al. Dramatically different phenotypic expressions of hypertrophic cardiomyopathy in male cousins undergoing cardiac transplantation with identical disease-causing gene mutation. Am J Cardiol [Internet]. 2013 Jun 15;111(12):1818–22. Available from: http://www.ncbi.nlm.nih.gov/pubmed/23540544
47. Wang C, Wu M, Qian J, Li B, Tu X, Xu C, et al. Identification of rare variants in TNNI3 with atrial fibrillation in a Chinese GeneID population. Mol Genet Genomics [Internet]. 2016 Feb;291(1):79–92. Available from: http://www.ncbi.nlm.nih.gov/pubmed/26169204
48. Sohn D-W, Kim H-K, Kim Y-J, Oh S, Seong M-W, Park S-S. Cardiomyopathies with Mixed and Inapparent Morphological Features in Cardiac Troponin I3 Mutation. Korean Circ J [Internet]. 2017 May;47(3):413–7. Available from: http://www.ncbi.nlm.nih.gov/pubmed/28567093
49. Calore C. Genotype-phenotype correlations and genetic family screening in hypertrophic cardiomyopathy. Università degli Studi di Padova; 2011.
50. Fujino M, Tsuda E, Hirono K, Nakata M, Ichida F, Hata Y, et al. The TNNI3 Arg192His mutation in a 13-year-old girl with left ventricular noncompaction. J Cardiol cases [Internet]. 2018 Jul;18(1):33–6. Available from: http://www.ncbi.nlm.nih.gov/pubmed/30279906
51. Chen Y, Yang S, Li J, Wang G, Qin Y, Wang D, et al. Pediatric restrictive cardiomyopathy due to a heterozygous mutation of the TNNI3 gene. J Biomed Res [Internet]. 2014 Jan;28(1):59–63. Available from: http://www.ncbi.nlm.nih.gov/pubmed/24474965

52. Rai TS, Ahmad S, Ahluwalia TS, Ahuja M, Bahl A, Saikia UN, et al. Genetic and clinical profile of Indian patients of idiopathic restrictive cardiomyopathy with and without hypertrophy. Mol Cell Biochem [Internet]. 2009 Nov;331(1–2):187–92. Available from: http://www.ncbi.nlm.nih.gov/pubmed/19449150

53. Otsuka H, Arimura T, Abe T, Kawai H, Aizawa Y, Kubo T, et al. Prevalence and distribution of sarcomeric gene mutations in Japanese patients with familial hypertrophic cardiomyopathy. Circ J [Internet]. 2012;76(2):453–61. Available from: http://www.ncbi.nlm.nih.gov/pubmed/22112859

54. Gambarin FI, Tagliani M, Arbustini E. Pure restrictive cardiomyopathy associated with cardiac troponin I gene mutation: mismatch between the lack of hypertrophy and the presence of disarray. Heart [Internet]. 2008 Oct;94(10):1257. Available from: http://www.ncbi.nlm.nih.gov/pubmed/18801787

55. Yang SW, Hitz M-P, Andelfinger G. Ventricular septal defect and restrictive cardiomyopathy in a paediatric TNNI3 mutation carrier. Cardiol Young [Internet]. 2010 Oct;20(5):574–6. Available from: http://www.ncbi.nlm.nih.gov/pubmed/20569525

56. Melacini P, Basso C, Angelini A, Calore C, Bobbo F, Tokajuk B, et al. Clinicopathological profiles of progressive heart failure in hypertrophic cardiomyopathy. Eur Heart J [Internet]. 2010 Sep;31(17):2111–23. Available from: http://www.ncbi.nlm.nih.gov/pubmed/20513729

57. Pinto JR, Siegfried JD, Parvatiyar MS, Li D, Norton N, Jones MA, et al. Functional characterization of TNNC1 rare variants identified in dilated cardiomyopathy. J Biol Chem [Internet]. 2011 Sep 30;286(39):34404–12. Available from: http://www.ncbi.nlm.nih.gov/pubmed/21832052

58. Landstrom AP, Parvatiyar MS, Pinto JR, Marquardt ML, Bos JM, Tester DJ, et al. Molecular and functional characterization of novel hypertrophic cardiomyopathy susceptibility mutations in TNNC1-encoded troponin C. J Mol Cell Cardiol [Internet]. 2008 Aug;45(2):281–8. Available from: http://www.ncbi.nlm.nih.gov/pubmed/18572189

59. Hoffmann B, Schmidt-Traub H, Perrot A, Osterziel KJ, Gessner R. First mutation in cardiac troponin C, L29Q, in a patient with hypertrophic cardiomyopathy. Hum Mutat [Internet]. 2001 Jun;17(6):524. Available from: http://www.ncbi.nlm.nih.gov/pubmed/11385718

60. Parvatiyar MS, Landstrom AP, Figueiredo-Freitas C, Potter JD, Ackerman MJ, Pinto JR. A mutation in TNNC1-encoded cardiac troponin C, TNNC1-A31S, predisposes to hypertrophic cardiomyopathy and ventricular fibrillation. J Biol Chem [Internet]. 2012 Sep 14;287(38):31845–55. Available from: http://www.ncbi.nlm.nih.gov/pubmed/22815480

61. van Spaendonck-Zwarts KY, van Tintelen JP, van Veldhuisen DJ, van der Werf R, Jongbloed JDH, Paulus WJ, et al. Peripartum cardiomyopathy as a part of familial dilated cardiomyopathy. Circulation [Internet]. 2010 May 25;121(20):2169–75. Available from: http://www.ncbi.nlm.nih.gov/pubmed/20458010

62. Lim CC, Yang H, Yang M, Wang C-K, Shi J, Berg EA, et al. A novel mutant cardiac troponin C disrupts molecular motions critical for calcium binding affinity and cardiomyocyte contractility. Biophys J [Internet]. 2008 May 1;94(9):3577–89. Available from: http://www.ncbi.nlm.nih.gov/pubmed/18212018

63. Nakajima-Taniguchi C, Matsui H, Fujio Y, Nagata S, Kishimoto T, Yamauchi-Takihara K. Novel missense mutation in cardiac troponin T gene found in Japanese patient with hypertrophic cardiomyopathy. J Mol Cell Cardiol [Internet]. 1997 Feb;29(2):839–43. Available from: http://www.ncbi.nlm.nih.gov/pubmed/9140840

64. Kaski JP, Burch M, Elliott PM. Mutations in the cardiac Troponin C gene are a cause of idiopathic dilated cardiomyopathy in childhood. Cardiol Young [Internet]. 2007 Dec;17(6):675–7. Available from: http://www.ncbi.nlm.nih.gov/pubmed/17977476

65. Watkins H, McKenna WJ, Thierfelder L, Suk HJ, Anan R, O'Donoghue A, et al. Mutations in the genes for cardiac troponin T and alpha-tropomyosin in hypertrophic cardiomyopathy. N Engl J Med [Internet]. 1995 Apr 20;332(16):1058–64. Available from: http://www.ncbi.nlm.nih.gov/pubmed/7898523
66. Pasquale F, Syrris P, Kaski JP, Mogensen J, McKenna WJ, Elliott P. Long-term outcomes in hypertrophic cardiomyopathy caused by mutations in the cardiac troponin T gene. Circ Cardiovasc Genet [Internet]. 2012 Feb 1;5(1):10–7. Available from: http://www.ncbi.nlm.nih.gov/pubmed/22144547
67. Thierfelder L, Watkins H, MacRae C, Lamas R, McKenna W, Vosberg HP, et al. Alpha-tropomyosin and cardiac troponin T mutations cause familial hypertrophic cardiomyopathy: a disease of the sarcomere. Cell [Internet]. 1994 Jun 3;77(5):701–12. Available from: http://www.ncbi.nlm.nih.gov/pubmed/8205619
68. Menon SC, Michels V V, Pellikka PA, Ballew JD, Karst ML, Herron KJ, et al. Cardiac troponin T mutation in familial cardiomyopathy with variable remodeling and restrictive physiology. Clin Genet [Internet]. 2008 Nov;74(5):445–54. Available from: http://www.ncbi.nlm.nih.gov/pubmed/18651846
69. Fujita E, Nakanishi T, Nishizawa T, Hagiwara N, Matsuoka R. Mutations in the cardiac troponin T gene show various prognoses in Japanese patients with hypertrophic cardiomyopathy. Heart Vessels [Internet]. 2013 Nov;28(6):785–94. Available from: http://www.ncbi.nlm.nih.gov/pubmed/23494605
70. Maron MS, Rowin EJ, Lin D, Appelbaum E, Chan RH, Gibson CM, et al. Prevalence and clinical profile of myocardial crypts in hypertrophic cardiomyopathy. Circ Cardiovasc Imaging [Internet]. 2012 Jul;5(4):441–7. Available from: http://www.ncbi.nlm.nih.gov/pubmed/22563033
71. Miller EM, Wang Y, Ware SM. Uptake of cardiac screening and genetic testing among hypertrophic and dilated cardiomyopathy families. J Genet Couns [Internet]. 2013 Apr;22(2):258–67. Available from: http://www.ncbi.nlm.nih.gov/pubmed/23054336
72. Frisso G, Limongelli G, Pacileo G, Del Giudice A, Forgione L, Calabrò P, et al. A child cohort study from southern Italy enlarges the genetic spectrum of hypertrophic cardiomyopathy. Clin Genet [Internet]. 2009 Jul;76(1):91–101. Available from: http://www.ncbi.nlm.nih.gov/pubmed/19659763
73. Konno T, Shimizu M, Ino H, Fujino N, Hayashi K, Uchiyama K, et al. Differences in diagnostic value of four electrocardiographic voltage criteria for hypertrophic cardiomyopathy in a genotyped population. Am J Cardiol [Internet]. 2005 Nov 1;96(9):1308–12. Available from: http://www.ncbi.nlm.nih.gov/pubmed/16253604
74. Gimeno JR, Monserrat L, Pérez-Sánchez I, Marín F, Caballero L, Hermida-Prieto M, et al. Hypertrophic cardiomyopathy. A study of the troponin-T gene in 127 Spanish families. Rev Esp Cardiol [Internet]. 2009 Dec;62(12):1473–7. Available from: http://www.ncbi.nlm.nih.gov/pubmed/20038417
75. Xu C, Wei M, Su B, Hua X-W, Zhang G-W, Xue X-P, et al. Ile90Met, a novel mutation in the cardiac troponin T gene for familial hypertrophic cardiomyopathy in a Chinese pedigree. Genet Res (Camb) [Internet]. 2008 Oct;90(5):445–50. Available from: http://www.ncbi.nlm.nih.gov/pubmed/19061534
76. Olivotto I, Girolami F, Ackerman MJ, Nistri S, Bos JM, Zachara E, et al. Myofilament protein gene mutation screening and outcome of patients with hypertrophic cardiomyopathy. Mayo Clin Proc [Internet]. 2008 Jun;83(6):630–8. Available from: http://www.ncbi.nlm.nih.gov/pubmed/18533079
77. Revera M, Van der Merwe L, Heradien M, Goosen A, Corfield VA, Brink PA, et al. Long-term follow-up of R403WMYH7 and R92WTNNT2 HCM families: mutations determine left ventricular dimensions but not wall thickness during disease progression.

Cardiovasc J Afr [Internet]. 18(3):146–53. Available from: http://www.ncbi.nlm.nih.gov/pubmed/17612745
78. Kassem HS, Azer RS, Saber-Ayad M, Ayad MS, Moharem-Elgamal S, Magdy G, et al. Early results of sarcomeric gene screening from the Egyptian National BA-HCM Program. J Cardiovasc Transl Res [Internet]. 2013 Feb;6(1):65–80. Available from: http://www.ncbi.nlm.nih.gov/pubmed/23233322
79. Marsiglia JDC, Credidio FL, de Oliveira TGM, Reis RF, Antunes M de O, de Araujo AQ, et al. Screening of MYH7, MYBPC3, and TNNT2 genes in Brazilian patients with hypertrophic cardiomyopathy. Am Heart J [Internet]. 2013 Oct;166(4):775–82. Available from: http://www.ncbi.nlm.nih.gov/pubmed/24093860
80. Fujino N, Shimizu M, Ino H, Okeie K, Yamaguchi M, Yasuda T, et al. Cardiac troponin T Arg92Trp mutation and progression from hypertrophic to dilated cardiomyopathy. Clin Cardiol [Internet]. 2001 May;24(5):397–402. Available from: http://www.ncbi.nlm.nih.gov/pubmed/11346248
81. Jáchymová M, Muravská A, Paleček T, Kuchynka P, Řeháková H, Magage S, et al. Genetic variation screening of TNNT2 gene in a cohort of patients with hypertrophic and dilated cardiomyopathy. Physiol Res [Internet]. 2012;61(2):169–75. Available from: http://www.ncbi.nlm.nih.gov/pubmed/22292720
82. Michels M, Soliman OII, Phefferkorn J, Hoedemaekers YM, Kofflard MJ, Dooijes D, et al. Disease penetrance and risk stratification for sudden cardiac death in asymptomatic hypertrophic cardiomyopathy mutation carriers. Eur Heart J [Internet]. 2009 Nov;30(21):2593–8. Available from: http://www.ncbi.nlm.nih.gov/pubmed/19666645
83. García-Castro M, Reguero JR, Morís C, Alonso-Montes C, Berrazueta JR, Sainz R, et al. Prevalence and spectrum of mutations in the sarcomeric troponin T and I genes in a cohort of Spanish cardiac hypertrophy patients. Int J Cardiol [Internet]. 2007 Sep 14;121(1):115–6. Available from: http://www.ncbi.nlm.nih.gov/pubmed/17101185
84. Torricelli F, Girolami F, Olivotto I, Passerini I, Frusconi S, Vargiu D, et al. Prevalence and clinical profile of troponin T mutations among patients with hypertrophic cardiomyopathy in tuscany. Am J Cardiol. 2003;92(11):1358–62.
85. Fujino N, Shimizu M, Ino H, Yamaguchi M, Yasuda T, Nagata M, et al. A novel mutation Lys273Glu in the cardiac troponin T gene shows high degree of penetrance and transition from hypertrophic to dilated cardiomyopathy. Am J Cardiol [Internet]. 2002 Jan 1;89(1):29–33. Available from: http://www.ncbi.nlm.nih.gov/pubmed/11779518
86. García-Castro M, Coto E, Reguero JR, Berrazueta JR, Alvarez V, Alonso B, et al. [Mutations in sarcomeric genes MYH7, MYBPC3, TNNT2, TNNI3, and TPM1 in patients with hypertrophic cardiomyopathy]. Rev Esp Cardiol [Internet]. 2009 Jan;62(1):48–56. Available from: http://www.ncbi.nlm.nih.gov/pubmed/19150014
87. Varnava A, Baboonian C, Davison F, de Cruz L, Elliott PM, Davies MJ, et al. A new mutation of the cardiac troponin T gene causing familial hypertrophic cardiomyopathy without left ventricular hypertrophy. Heart [Internet]. 1999 Nov;82(5):621–4. Available from: http://www.ncbi.nlm.nih.gov/pubmed/10525521
88. Luedde M, Ehlermann P, Weichenhan D, Will R, Zeller R, Rupp S, et al. Severe familial left ventricular non-compaction cardiomyopathy due to a novel troponin T (TNNT2) mutation. Cardiovasc Res [Internet]. 2010 Jun 1;86(3):452–60. Available from: http://www.ncbi.nlm.nih.gov/pubmed/20083571
89. Maron BJ, Yeates L, Semsarian C. Clinical challenges of genotype positive (+)-phenotype negative (-) family members in hypertrophic cardiomyopathy. Am J Cardiol [Internet]. 2011 Feb 15;107(4):604–8. Available from: http://www.ncbi.nlm.nih.gov/pubmed/21185001

90. Anan R, Shono H, Kisanuki A, Arima S, Nakao S, Tanaka H. Patients with familial hypertrophic cardiomyopathy caused by a Phe110Ile missense mutation in the cardiac troponin T gene have variable cardiac morphologies and a favorable prognosis. Circulation [Internet]. 1998 Aug 4;98(5):391–7. Available from: http://www.ncbi.nlm.nih.gov/pubmed/9714088
91. Lin T-L, Ichihara S, Yamada Y, Nagasaka T, Ishihara H, Nakashima N, et al. Phenotypic Variation of Familial Hypertrophic Cardiomyopathy Caused by the Phe110→Ile Mutation in Cardiac Troponin T. Cardiology [Internet]. 2000;93(3):155–62. Available from: http://www.ncbi.nlm.nih.gov/pubmed/10965086
92. Song L, Zou Y, Wang J, Wang Z, Zhen Y, Lou K, et al. Mutations profile in Chinese patients with hypertrophic cardiomyopathy. Clin Chim Acta [Internet]. 2005 Jan;351(1–2):209–16. Available from: http://www.ncbi.nlm.nih.gov/pubmed/15563892
93. Probst S, Oechslin E, Schuler P, Greutmann M, Boyé P, Knirsch W, et al. Sarcomere gene mutations in isolated left ventricular noncompaction cardiomyopathy do not predict clinical phenotype. Circ Cardiovasc Genet [Internet]. 2011 Aug 1;4(4):367–74. Available from: http://www.ncbi.nlm.nih.gov/pubmed/21551322
94. Morales A, Pinto JR, Siegfried JD, Li D, Norton N, Hofmeyer M, et al. Late onset sporadic dilated cardiomyopathy caused by a cardiac troponin T mutation. Clin Transl Sci [Internet]. 2010 Oct;3(5):219–26. Available from: http://www.ncbi.nlm.nih.gov/pubmed/20973921
95. Hershberger RE, Pinto JR, Parks SB, Kushner JD, Li D, Ludwigsen S, et al. Clinical and functional characterization of TNNT2 mutations identified in patients with dilated cardiomyopathy. Circ Cardiovasc Genet [Internet]. 2009 Aug;2(4):306–13. Available from: http://www.ncbi.nlm.nih.gov/pubmed/20031601
96. Li D, Czernuszewicz GZ, Gonzalez O, Tapscott T, Karibe A, Durand JB, et al. Novel cardiac troponin T mutation as a cause of familial dilated cardiomyopathy. Circulation [Internet]. 2001 Oct 30;104(18):2188–93. Available from: http://www.ncbi.nlm.nih.gov/pubmed/11684629
97. Rani DS, Dhandapany PS, Nallari P, Narasimhan C, Thangaraj K. A novel arginine to tryptophan (R144W) mutation in troponin T (cTnT) gene in an indian multigenerational family with dilated cardiomyopathy (FDCM). PLoS One [Internet]. 2014;9(7):e101451. Available from: http://www.ncbi.nlm.nih.gov/pubmed/24992688
98. Morales A, Painter T, Li R, Siegfried JD, Li D, Norton N, et al. Rare variant mutations in pregnancy-associated or peripartum cardiomyopathy. Circulation [Internet]. 2010 May 25;121(20):2176–82. Available from: http://www.ncbi.nlm.nih.gov/pubmed/20458009
99. Stefanelli CB, Rosenthal A, Borisov AB, Ensing GJ, Russell MW. Novel troponin T mutation in familial dilated cardiomyopathy with gender-dependant severity. Mol Genet Metab [Internet]. 83(1–2):188–96. Available from: http://www.ncbi.nlm.nih.gov/pubmed/15464434
100. Campbell N, Sinagra G, Jones KL, Slavov D, Gowan K, Merlo M, et al. Whole exome sequencing identifies a troponin T mutation hot spot in familial dilated cardiomyopathy. PLoS One [Internet]. 2013;8(10):e78104. Available from: http://www.ncbi.nlm.nih.gov/pubmed/24205113
101. Van Acker H, De Sutter J, Vandekerckhove K, de Ravel TJL, Verhaaren H, De Backer J. Dilated cardiomyopathy caused by a novel TNNT2 mutation-added value of genetic testing in the correct identification of affected subjects. Int J Cardiol [Internet]. 2010 Oct 8;144(2):307–9. Available from: http://www.ncbi.nlm.nih.gov/pubmed/19324435
102. Ho CY, Lever HM, DeSanctis R, Farver CF, Seidman JG, Seidman CE. Homozygous mutation in cardiac troponin T: implications for hypertrophic cardiomyopathy. Circulation

[Internet]. 2000 Oct 17;102(16):1950–5. Available from: http://www.ncbi.nlm.nih.gov/pubmed/11034944
103. Hirtle-Lewis M, Desbiens K, Ruel I, Rudzicz N, Genest J, Engert JC, et al. The genetics of dilated cardiomyopathy: a prioritized candidate gene study of LMNA, TNNT2, TCAP, and PLN. Clin Cardiol [Internet]. 2013 Oct;36(10):628–33. Available from: http://www.ncbi.nlm.nih.gov/pubmed/24037902
104. Miliou A, Anastasakis A, D'Cruz LG, Theopistou A, Rigopoulos A, Rizos I, et al. Low prevalence of cardiac troponin T mutations in a Greek hypertrophic cardiomyopathy cohort. Heart [Internet]. 2005 Jul;91(7):966–7. Available from: http://www.ncbi.nlm.nih.gov/pubmed/15958377
105. Theopistou A, Anastasakis A, Miliou A, Rigopoulos A, Toutouzas P, Stefanadis C. Clinical features of hypertrophic cardiomyopathy caused by an Arg278Cys missense mutation in the cardiac troponin T gene. Am J Cardiol [Internet]. 2004 Jul 15;94(2):246–9. Available from: http://www.ncbi.nlm.nih.gov/pubmed/15246915
106. Narsupalli S, Castle B, Veldtman G. Resolution of left ventricular and asymmetric septal hypertrophy after resection of left ventricular outflow obstruction in a patient with troponin-positive hypertrophic obstructive cardiomyopathy: a case report. Cardiol Young [Internet]. 2010 Oct;20(5):580–3. Available from: http://www.ncbi.nlm.nih.gov/pubmed/20663266
107. Bottillo I, D'Angelantonio D, Caputo V, Paiardini A, Lipari M, De Bernardo C, et al. Molecular analysis of sarcomeric and non-sarcomeric genes in patients with hypertrophic cardiomyopathy. Gene [Internet]. 2016 Feb 15;577(2):227–35. Available from: http://www.ncbi.nlm.nih.gov/pubmed/26656175
108. Rubattu S, Bozzao C, Pennacchini E, Pagannone E, Musumeci BM, Piane M, et al. A Next-Generation Sequencing Approach to Identify Gene Mutations in Early- and Late-Onset Hypertrophic Cardiomyopathy Patients of an Italian Cohort. Int J Mol Sci [Internet]. 2016 Jul 30;17(8). Available from: http://www.ncbi.nlm.nih.gov/pubmed/27483260
109. Lopes LR, Syrris P, Guttmann OP, O'Mahony C, Tang HC, Dalageorgou C, et al. Novel genotype-phenotype associations demonstrated by high-throughput sequencing in patients with hypertrophic cardiomyopathy. Heart [Internet]. 2015 Feb;101(4):294–301. Available from: http://www.ncbi.nlm.nih.gov/pubmed/25351510
110. Ho CY, Carlsen C, Thune JJ, Havndrup O, Bundgaard H, Farrohi F, et al. Echocardiographic strain imaging to assess early and late consequences of sarcomere mutations in hypertrophic cardiomyopathy. Circ Cardiovasc Genet [Internet]. 2009 Aug;2(4):314–21. Available from: http://www.ncbi.nlm.nih.gov/pubmed/20031602
111. Møller DV, Andersen PS, Hedley P, Ersbøll MK, Bundgaard H, Moolman-Smook J, et al. The role of sarcomere gene mutations in patients with idiopathic dilated cardiomyopathy. Eur J Hum Genet [Internet]. 2009 Oct;17(10):1241–9. Available from: http://www.ncbi.nlm.nih.gov/pubmed/19293840
112. Lu C, Wu W, Liu F, Yang K, Li J, Liu Y, et al. Molecular analysis of inherited cardiomyopathy using next generation semiconductor sequencing technologies. J Transl Med [Internet]. 2018;16(1):241. Available from: http://www.ncbi.nlm.nih.gov/pubmed/30165862
113. Zhao Y, Feng Y, Zhang Y-M, Ding X-X, Song Y-Z, Zhang A-M, et al. Targeted next-generation sequencing of candidate genes reveals novel mutations in patients with dilated cardiomyopathy. Int J Mol Med [Internet]. 2015 Dec;36(6):1479–86. Available from: http://www.ncbi.nlm.nih.gov/pubmed/26458567
114. Mademont-Soler I, Mates J, Yotti R, Espinosa MA, Pérez-Serra A, Fernandez-Avila AI, et al. Additional value of screening for minor genes and copy number variants in hypertrophic cardiomyopathy. PLoS One [Internet]. 2017;12(8):e0181465. Available from: http://www.ncbi.nlm.nih.gov/pubmed/28771489

115. Gomes A V, Potter JD. Cellular and molecular aspects of familial hypertrophic cardiomyopathy caused by mutations in the cardiac troponin I gene. Mol Cell Biochem [Internet]. 2004;263(1–2):99–114. Available from: http://www.ncbi.nlm.nih.gov/pubmed/15524171
116. Mouton JM, Pellizzon AS, Goosen A, Kinnear CJ, Herbst PG, Brink PA, et al. Diagnostic disparity and identification of two TNNI3 gene mutations, one novel and one arising de novo, in South African patients with restrictive cardiomyopathy and focal ventricular hypertrophy. Cardiovasc J Afr [Internet]. 26(2):63–9. Available from: http://www.ncbi.nlm.nih.gov/pubmed/25940119
117. Lesizza P, Merlo M, Vitrella G, Sinagra G. Hypertrophic restrictive cardiomyopathy with apical thinning: a peculiar case of genotype-phenotype correlation. J Cardiovasc Med (Hagerstown) [Internet]. 2017;18(10):835–6. Available from: http://www.ncbi.nlm.nih.gov/pubmed/28650931
118. Hazebroek MR, Krapels I, Verdonschot J, van den Wijngaard A, Vanhoutte E, Hoos M, et al. Prevalence of Pathogenic Gene Mutations and Prognosis Do Not Differ in Isolated Left Ventricular Dysfunction Compared With Dilated Cardiomyopathy. Circ Heart Fail [Internet]. 2018 Mar;11(3):e004682. Available from: http://www.ncbi.nlm.nih.gov/pubmed/29540472
119. Pan S, Sommese RF, Sallam KI, Nag S, Sutton S, Miller SM, et al. Establishing disease causality for a novel gene variant in familial dilated cardiomyopathy using a functional in-vitro assay of regulated thin filaments and human cardiac myosin. BMC Med Genet [Internet]. 2015 Oct 26;16:97. Available from: http://www.ncbi.nlm.nih.gov/pubmed/264985

**Supplementary Table 3. Structures of thin filament protein components and complexes.**

| PDB | Description | Protein | Species | Sequence[a] | Human cardiac sequence[a] |
|---|---|---|---|---|---|
| 1J1E[b] | Troponin ($Ca^{2+}$-bound) | Cardiac TnC | Human | P63316:1-161 | P63316:1-161 |
| | | Cardiac TnT | Human | P45379:212-286 | P45379:212-286 |
| | | Cardiac TnI | Human | P19429:35-162 | P19429:35-162 |
| 1YTZ[c] | Troponin ($Ca^{2+}$-bound) | Skeletal TnC | Chicken | P02588:2-163 | P63316:2-161 |
| | | Skeletal TnT | Chicken | P12620:160-249 | P45379:194-285 |
| | | Skeletal TnI | Chicken | P68246:4-144 | P19429:35-176 |
| 1YV0[c] | Troponin ($Ca^{2+}$-free) | Skeletal TnC | Chicken | P02588:2-163 | P63316:2-161 |
| | | Skeletal TnT | Chicken | P12620:160-249 | P45379:194-285 |
| | | Skeletal TnI | Chicken | P68246:4-119 | P19429:35-151 |
| 2W4U[d] | Troponin, tropomyosin and actin | Skeletal TnC | Chicken | P02588:5-163 | P63316:5-161 |
| | | Skeletal TnT | Chicken | P12620:160-249 | P45379:194-285 |
| | | Skeletal TnI | Chicken | P68246:4-144 | P19429:35-176 |
| | | Tropomyosin | Rabbit | P58772:8-284 | P09493:8-284 |

| | | | | | |
|---|---|---|---|---|---|
| | | Skeletal actin | Rabbit | P68135:3-374 | P68032:3-374 |
| 2Z5H[e] | Tropomyosin & TnT | Tropomyosin | Rabbit | P58772:254-284 | P09493:254-284 |
| | | Skeletal TnT | Chicken | P12520:58-112 | P45379:100-133 |
| 1VDI[f] | TnI ($Ca^{2+}$-bound) | Skeletal TnI | Chicken | P68246:132-183 | P19429:164-210 |
| 1VDJ[f] | TnI ($Ca^{2+}$-free) | Skeletal TnI | Chicken | P68246:132-183 | P19429:164-210 |

[a] UniProt identifier and sequence range resolved in structure. [b] [PMID:12840750]; [c] [PMID:15784741]; [d] [PMID:22761792]; [e] [PMID:18483193]; [f] [PMID:16061251]

**Supplementary Table 4.** Estimated penetrance and odds risk calculator in a case-control study of patients harbouring the variants p.Arg278Cys and p.Arg286His in TNNT2.

| Variant | HCM Case frequency (variants/total) | GnomAD freq. (variants/total) | Odds ratio | Estimated penetrance | Disease controls freq. | Odds ratio | Estimated penetrance |
|---|---|---|---|---|---|---|---|
| **p.Arg278 Cys** | 0.71% (62/8,696) | 0.071% (98/137,785) | **10.1** (7.33-13.1) | **2.0%** (1.3-3.1) | 0.086% (9/10,364) | **4.85** (3.46-6.80) | **1.6%** (0.07-4.0) |
| **p.Arg286 His** | 0.16% (17/8,696) | 0.01% (19/136,988) | **14.4** (7.4-27.2) | **2.8%** (1.1-7) | 0.02% (2/10,364) | **10.14** (2.34-43.9) | **2.0%** (0.35-12) |

Enrichment of variations p.Arg278Cys and p.Arg286 His in HCM probands compared to controls in the HIC cohort (20,713 probands with different inherited cardiac conditions). Variations are clearly enriched in HCM cohort, with an OR > 5. This adds to these variant the PS4 criteria (strong) that supports the classification of the variations as likely pathogenic.

Penetrance was estimated according the study of Minkel et al. Sci Transl Med. 2016 Jan 20;8(322):322ra9.

**Supplementary Figure 4. Ramachandran plot of the cTnT wildtype model 1.**

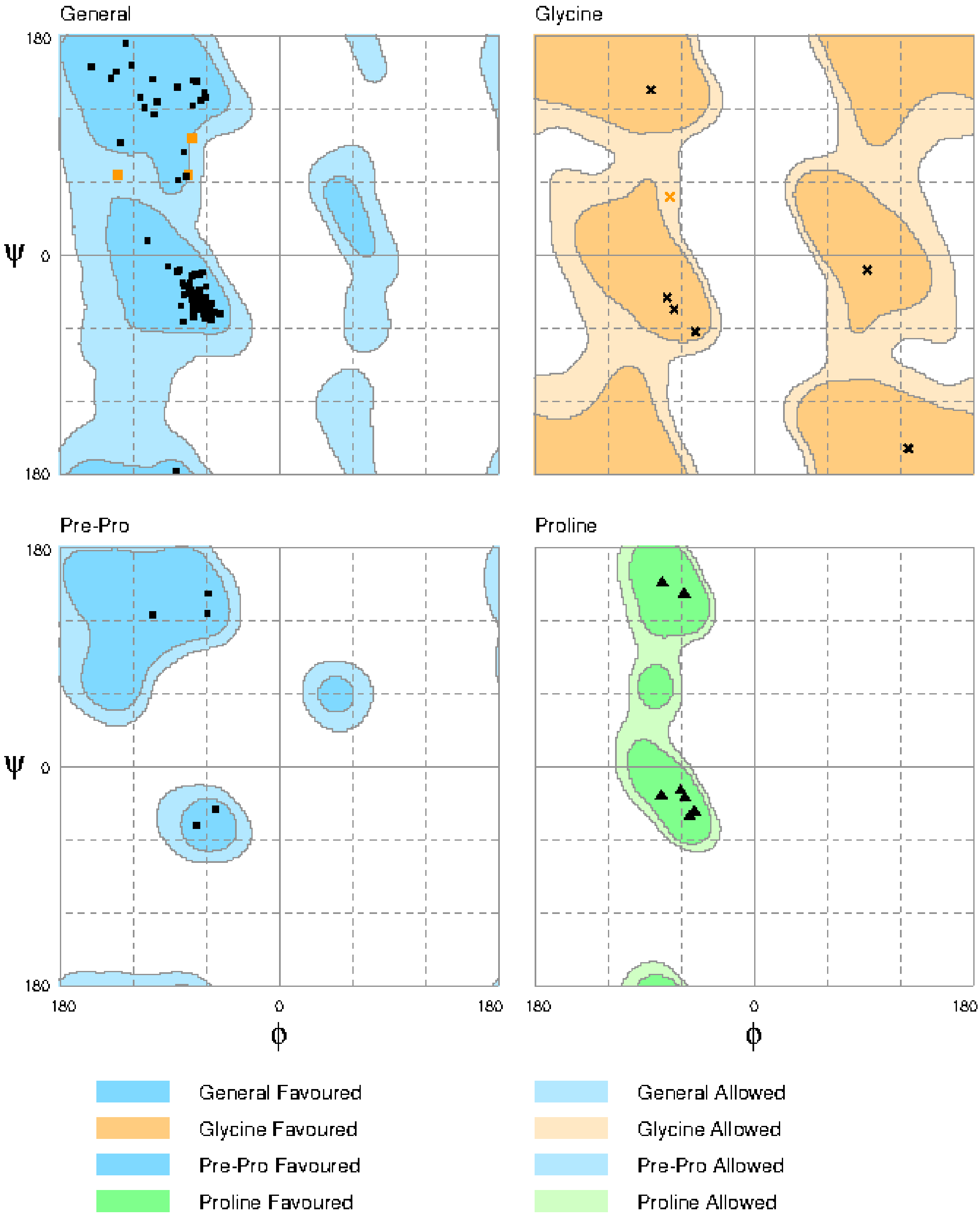


Number of residues in favoured region (~98.0% expected) : 292 (98.6%)
Number of residues in allowed region (~2.0% expected) : 4 (1.4%)
Number of residues in outlier region : 0 (0.0%)

**Supplementary Figure 5. Ramachandran plot of the cTnT wildtype model 2.**

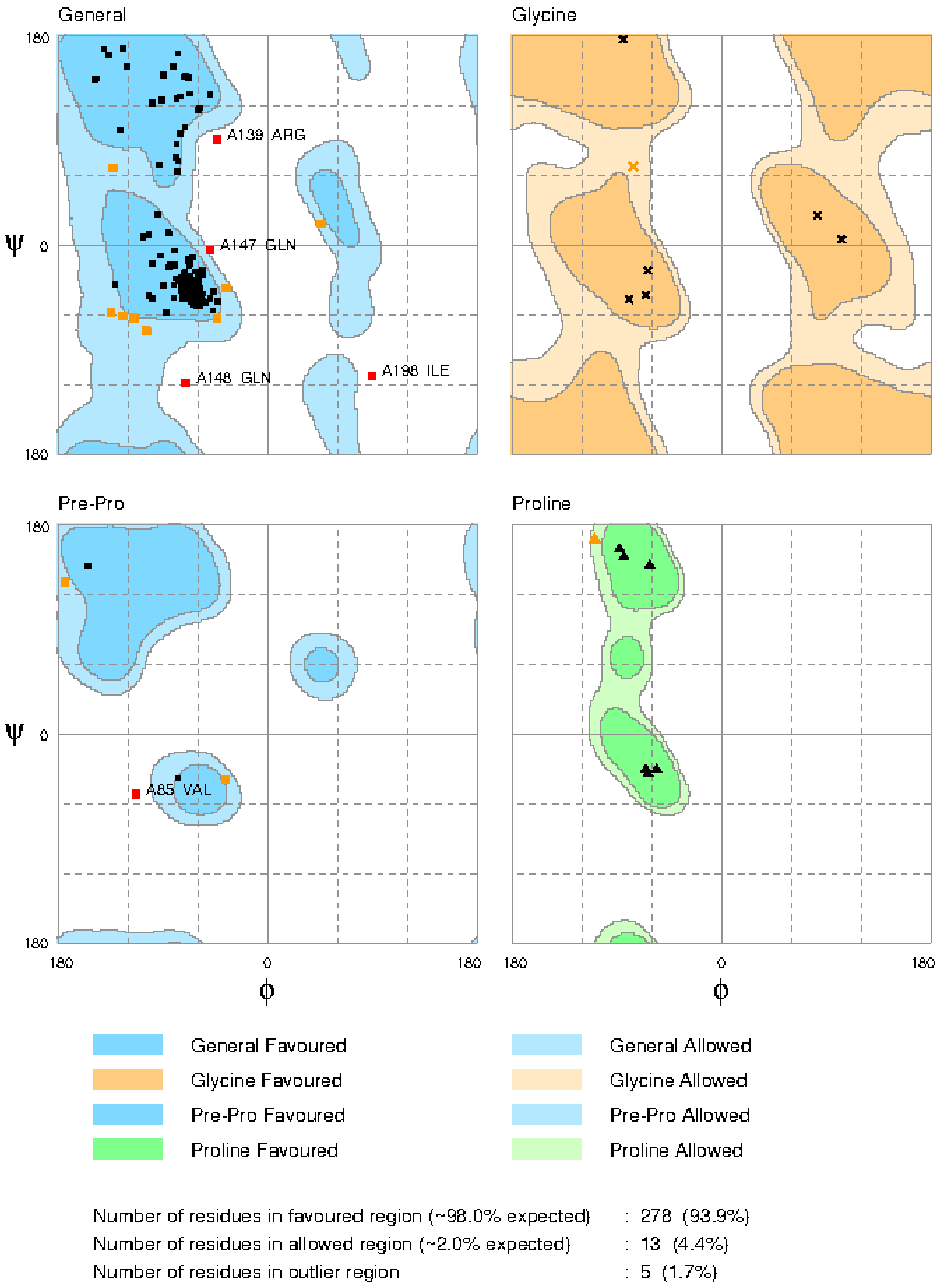

**Supplementary Figure 6. Ramachandran plot of the cTnT WT model 3.**

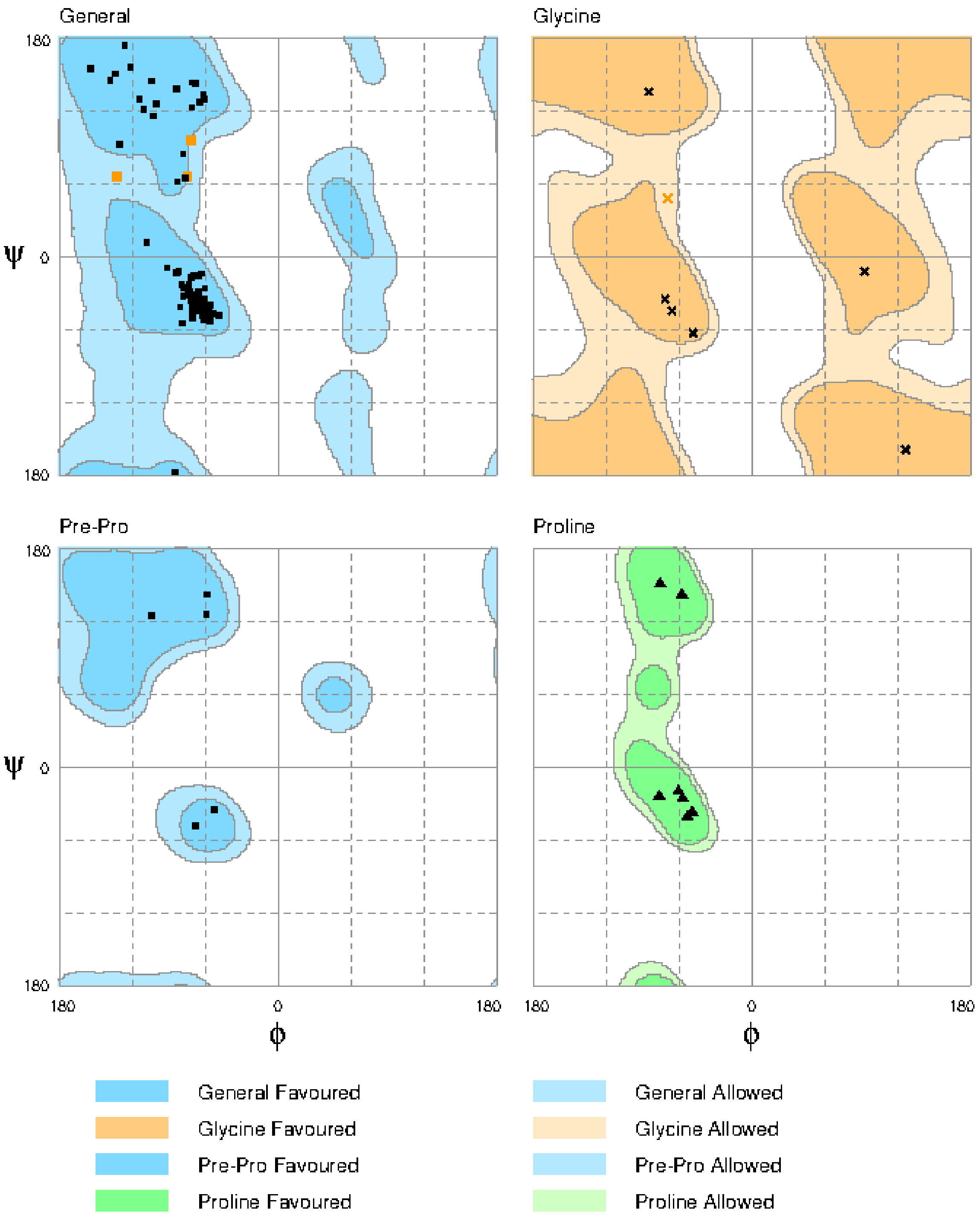


| | | |
|---|---|---|
| Number of residues in favoured region (~98.0% expected) | : | 292 (98.6%) |
| Number of residues in allowed region (~2.0% expected) | : | 4 (1.4%) |
| Number of residues in outlier region | : | 0 (0.0%) |

**Supplementary Figure 7. Ramachandran plot of the cTnT wildtype model 4.**

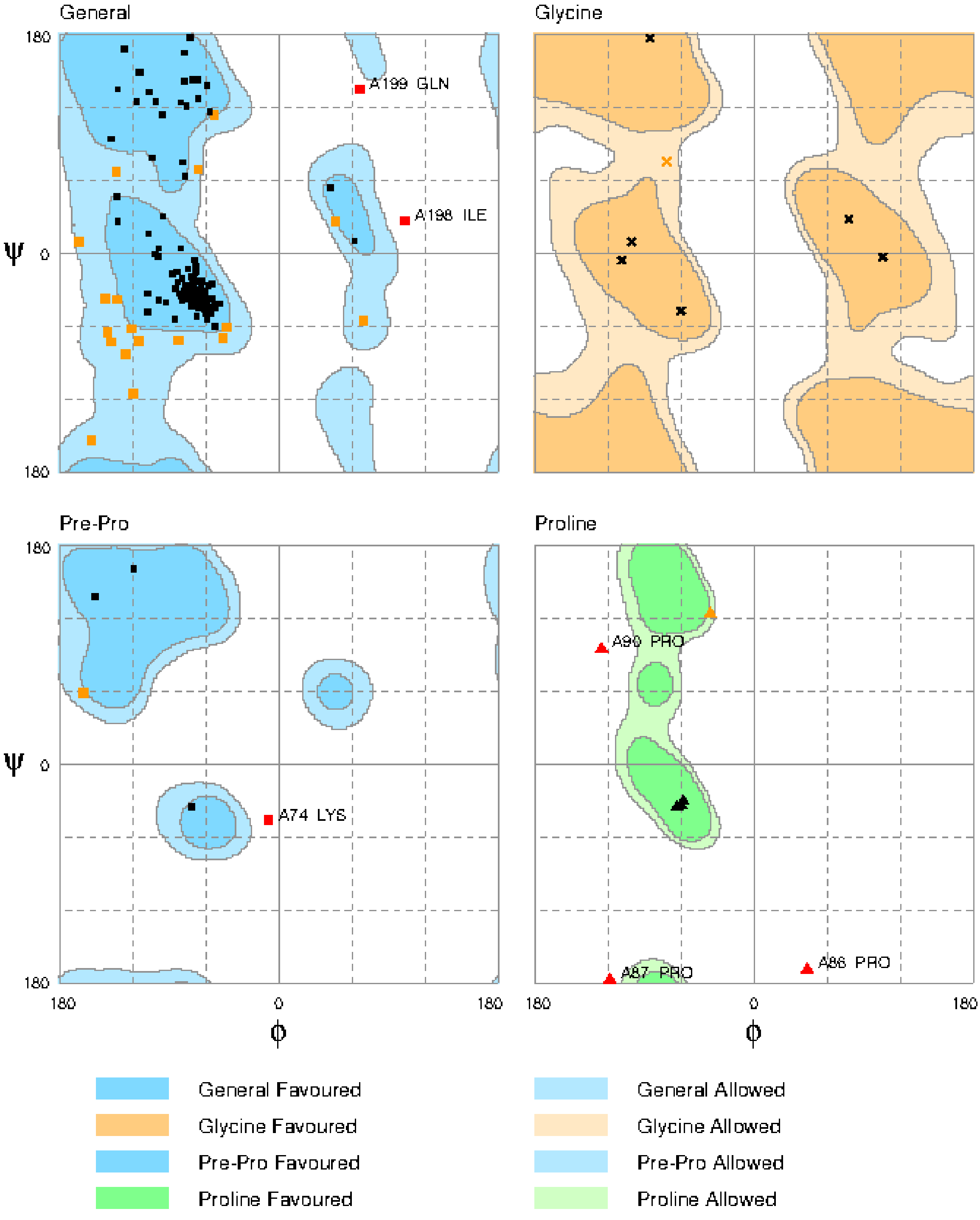


Number of residues in favoured region (~98.0% expected) : 269 (90.9%)
Number of residues in allowed region (~2.0% expected) : 21 (7.1%)
Number of residues in outlier region : 6 (2.0%)

**Supplementary Figure 8. Ramachandran plot of the cTnT wildtype model 5.**

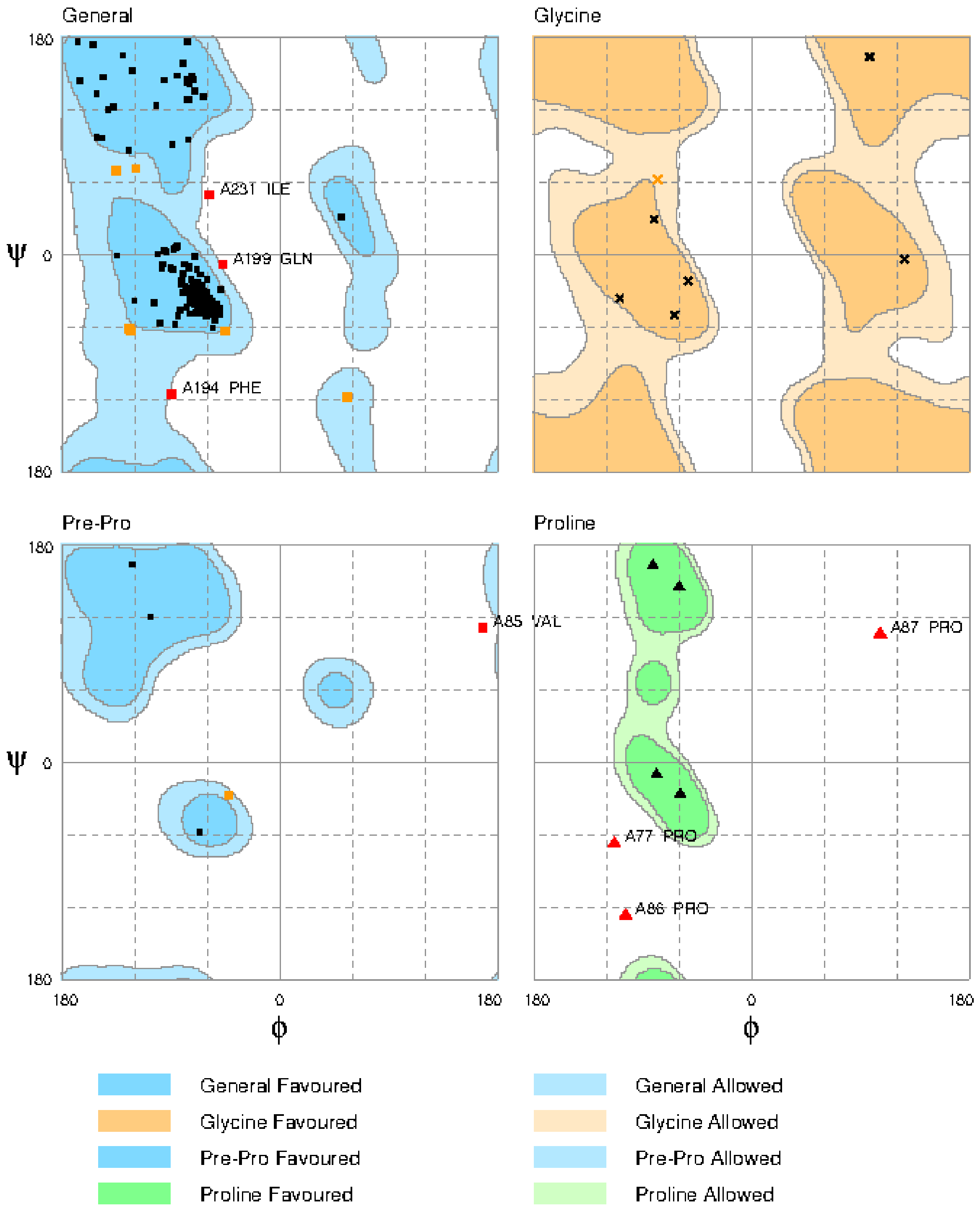


Number of residues in favoured region (~98.0% expected) : 281 (94.9%)
Number of residues in allowed region (~2.0% expected) : 8 (2.7%)
Number of residues in outlier region : 7 (2.4%)

**Supplementary Figure 9.** Comparison of the survival from cardiovascular death in carriers of variants in TNNI3 according to regions.

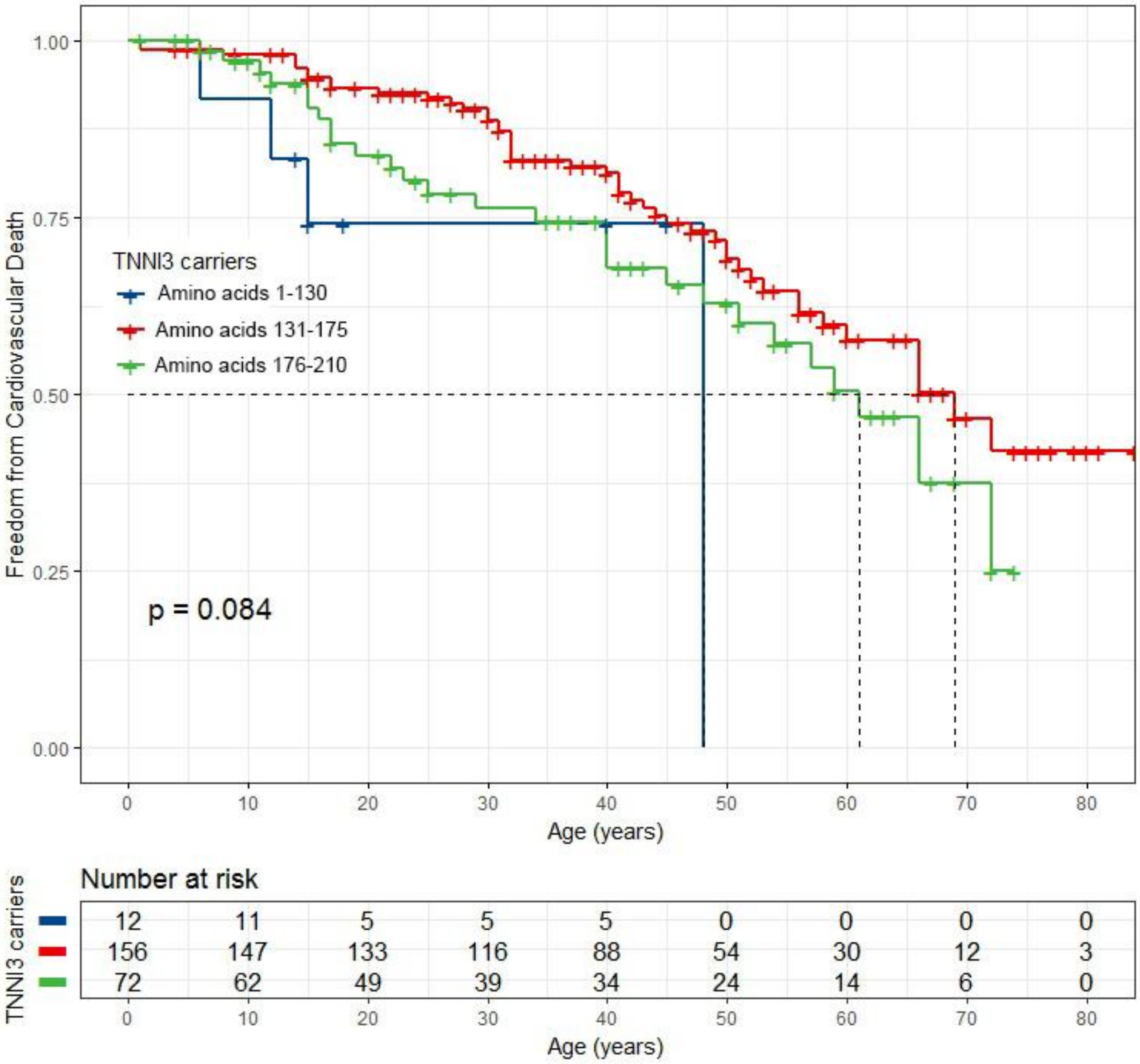


Comparison of the regions involving amino acids 1-130 (blue), 131-175 (red), and 176-210 (green). There were no statistically significant differences between the three regions in terms of survival of cardiovascular death (p=0.084), but data were scarce for patients in region 1-

130.

Pairwise comparison between regions 131-275 and 176-210 showed a worst survival for the later (p=0.008), with medians of survival of 61 and 69 years respectively.

**Supplementary Movie 1. Dominant motions in WT cTnT.**

https://drive.google.com/file/d/1kIR6M8bZhCe0ClCQeR2X_8Fu6_McD6GM/view

**Supplementary Movie 2. Dominant motions in R102Q cTnT.**

https://drive.google.com/file/d/1D_quD1E-2kjaU4KYTFU3JOTW4LlT7qWa/view

**Supplementary Movie 3. Dominant motions in R102W cTnT.**

https://drive.google.com/file/d/1C-g1lPhqJQBZ1Qy5cgrEfYsryWIpUTGp/view

**Supplementary Movie 4. Dominant motions in R141W cTnT.**

https://drive.google.com/file/d/1HfQbantc04nqAZsItVMP53b9fNLZDjUF/view

**Supplementary Movie 5. Dominant motions in R173W cTnT.**

https://drive.google.com/file/d/1meA85SIjSxMXwb6pYjiNK5Smez6qHYCb/view
