# Supplementary material for: Prognostic implications of troponin T variations in inherited cardiomyopathies using systems biology
Source: NPJ Genom Med. 2021 Jun 14;6:47. doi: 10.1038/s41525-021-00204-w (PMC8203786; doi:10.1038/s41525-021-00204-w)
Supplement: Supplementary file 2 — Reporting Summary [file 41525_2021_204_MOESM2_ESM.pdf]

nature research

Corresponding author(s): Lorenzo Monserrat & Rameen Shakur

Reporting Summary

Nature Research wishes to improve the reproducibility of the work that we publish. This form provides structure for consistency and transparency in reporting. For further information on Nature Research policies, see Authors & Referees and the Editorial Policy Checklist.

Statistical parameters

When statistical analyses are reported, confirm that the following items are present in the relevant location (e.g. figure legend, table legend, main text, or Methods section).

n/a	Confirmed	
☒	☐	The exact sample size (*n*) for each experimental group/condition, given as a discrete number and unit of measurement
☒	☐	An indication of whether measurements were taken from distinct samples or whether the same sample was measured repeatedly
☐	☒	The statistical test(s) used AND whether they are one- or two-sided<br>*Only common tests should be described solely by name; describe more complex techniques in the Methods section.*
☒	☐	A description of all covariates tested
☒	☐	A description of any assumptions or corrections, such as tests of normality and adjustment for multiple comparisons
☐	☒	A full description of the statistics including central tendency (e.g. means) or other basic estimates (e.g. regression coefficient) AND variation (e.g. standard deviation) or associated estimates of uncertainty (e.g. confidence intervals)
☐	☒	For null hypothesis testing, the test statistic (e.g. *F*, *t*, *r*) with confidence intervals, effect sizes, degrees of freedom and *P* value noted<br>*Give P values as exact values whenever suitable.*
☒	☐	For Bayesian analysis, information on the choice of priors and Markov chain Monte Carlo settings
☒	☐	For hierarchical and complex designs, identification of the appropriate level for tests and full reporting of outcomes
☒	☐	Estimates of effect sizes (e.g. Cohen's *d*, Pearson's *r*), indicating how they were calculated
☒	☐	Clearly defined error bars<br>*State explicitly what error bars represent (e.g. SD, SE, CI)*

Our web collection on statistics for biologists may be useful.

Software and code

Policy information about availability of computer code

Data collection	n/a
Data analysis	n/a

For manuscripts utilizing custom algorithms or software that are central to the research but not yet described in published literature, software must be made available to editors/reviewers upon request. We strongly encourage code deposition in a community repository (e.g. GitHub). See the Nature Research guidelines for submitting code & software for further information.

Data

Policy information about availability of data

All manuscripts must include a data availability statement. This statement should provide the following information, where applicable:

- Accession codes, unique identifiers, or web links for publicly available datasets
- A list of figures that have associated raw data
- A description of any restrictions on data availability

We undertook a systematic review and literature search and applied this to our model . We stated the methodology , with full exclusion and inclusion criteria etc in the paper and moreover referenced all the papers in an additional table of supplementary references for readers to access just as we had used for our analysis and which were utilized for integration on our model.

Field-specific reporting

Please select the best fit for your research. If you are not sure, read the appropriate sections before making your selection.

☒ Life sciences ☐ Behavioural & social sciences ☐ Ecological, evolutionary & environmental sciences

For a reference copy of the document with all sections, see nature.com/authors/policies/ReportingSummary-flat.pdf

Life sciences study design

All studies must disclose on these points even when the disclosure is negative.

Sample size

Ours was a systematic review and so we performed a comprehensive search of PubMed articles (1st January 1971 to 1st November 2019) to collect clinical information of families and individuals who carry amino acid substitutions in cardiac troponins associated with cardiomyopathy

Data exclusions

Exclusion and Inclusion criteria for our review are stipulated in the paper :

The search terms used were:
“English”[Language] AND
(“1900/01/01”[Date - Publication]: “2019/11/31” [Date - Publication]) AND
("hypertrophic subaortic stenosis" OR
HSS* OR
"muscular subaortic stenosis" OR
"asymmetric septal hypertrophy" OR
ASH OR
"asymmetric septal hypertrophy" OR
"hypertrophic cardiomyopathy" OR
"hypertrophic obstructive cardiomyopathy" OR
"hypertrophic cardiomyopathies" OR
HCM OR
("dilated cardiomyopathy") OR
("restrictive cardiomyopathy" OR "restrictive cardiomyopathies")) AND
("troponin T type 2" OR "troponin T" OR TNNT2 OR “troponin C” OR TNNC1 OR "troponin I" OR TNNI3 OR troponins)

Titles and abstracts of the identified articles were evaluated by two experts. Next, the full article texts were evaluated and those meeting the following criteria were selected: observational English language reports describing phenotypic features (HCM; DCM; restrictive cardiomyopathy; left ventricular non-compaction) in patients with variations in genes TNNC1, TNNT2 or TNNI3; and studies published in peer-reviewed journals. In addition, a manual search of the reference lists of the identified studies was performed, and references were evaluated using the same inclusion and exclusion criteria. Studies were included if they had information on relatives.
The collected information were stored into a database which includes for each study: country of origin (ISO country names); patient age at diagnosis, and last follow-up age; family history of cardiomyopathy; morphology and function of the heart evaluated by cardiac imaging (extent and pattern of hypertrophy, late gadolinium enhancement, atrial and ventricular dimensions and function, left ventricular outflow tract obstruction, mitral valve abnormalities); clinical risk factors for SCD (maximum left ventricular wall thickness ≥30 mm, abnormal exercise blood pressure response, non-sustained ventricular tachycardia, family history of SCD, syncope); interventions, outcome and prognosis (all death, cardiovascular death, SCD, non-fatal HF, AF, non-fatal stroke, implantable cardioverter-defibrillator (ICD) implantation, myectomy, alcohol septal ablation).
All the variants were classified according to ACMG (American Collage of Medical Genetics and Genomics)31 guidelines for the interpretation of sequence variants. We considered the presence of the variant in control databases; number of studies and descriptive families; functional studies; evidence of co-segregation of the variant with the phenotype; and computational evidence support. A variant was considered pathogenic if it had causative variations in at least three independent peer-reviewed studies with non-contradictory evidence. If the evidence for pathogenicity was contradictory, internal information of more than 20,000 patients sequenced in Health In Code Database was used for support or reject pathogenicity for rare variants (i.e. case-control analysis was performed to define pathogenicity of variations in amino acids p.Arg278 and p.Arg286; Supplementary Table 4.). Every variant was reviewed by cardiologists specialized in genetics (L.M., J.P.O.) who evaluated the evidence to confirm the variant classification. Only variants classified as pathogenic or likely pathogenic for cardiomyopathy were included.

Replication

review of all data sets and review of clinical data sets was checked by 2 clinicians and data input for each paper checked and verified

Randomization

not applicable in our study

Blinding

Blinding was done in 2 phases: In our unbiased model the team developing the integrative molecular model was blinded to the data search and the clinical team who reviewed and analyzed the clinical data sets, this team were also blinded to the molecular model initial model . Once both teams had completed there initial points of investigation both data sets were integrated. All statistical analysis was done independently by a member blinded to the model and the outcomes.

Reporting for specific materials, systems and methods

Materials & experimental systems

n/a	Involved in the study
☒	☐ Unique biological materials
☒	☐ Antibodies
☒	☐ Eukaryotic cell lines
☒	☐ Palaeontology
☒	☐ Animals and other organisms
☒	☐ Human research participants

Methods

n/a	Involved in the study
☒	☐ ChIP-seq
☒	☐ Flow cytometry
☒	☐ MRI-based neuroimaging
