# Supplementary material for: Prognostic implications of troponin T variations in inherited cardiomyopathies using systems biology
Source: NPJ Genom Med. 2021 Jun 14;6:47. doi: 10.1038/s41525-021-00204-w (PMC8203786; doi:10.1038/s41525-021-00204-w)
Supplement: Supplementary file 3 — Supplementary Movie titles [file 41525_2021_204_MOESM3_ESM.pdf]

Supplementary Movies

1)Supplementary Movie 1. Dominant motions in WT cTnT.

2)Supplementary Movie 2. Dominant motions in R102Q cTnT.

3)Supplementary Movie 3. Dominant motions in R102W cTnT.

4)Supplementary Movie 4. Dominant motions in R141W cTnT.

5)Supplementary Movie 5. Dominant motions in R173W cTnT.
